# castleCSF — A contrast sensitivity function of color, area, spatiotemporal frequency, luminance and eccentricity

**DOI:** 10.1167/jov.24.4.5

**Published:** 2024-04-04

**Authors:** Maliha Ashraf, Rafał K. Mantiuk, Alexandre Chapiro, Sophie Wuerger

**Affiliations:** 1 Department of Computer Science and Technology University of Cambridge, Cambridge, UK; 2 Department of Computer Science and Technology University of Cambridge, Cambridge, UK; 3 Applied Perception Science Group Meta, Sunnyvale, CA, USA; 4 Department of Psychology University of Liverpool, Liverpool, UK

**Keywords:** contrast sensitivity functions, color vision, computational modeling

## Abstract

The contrast sensitivity function (CSF) is a fundamental visual model explaining our ability to detect small contrast patterns. CSFs found many applications in engineering, where they can be used to optimize a design for perceptual limits. To serve such a purpose, CSFs must explain possibly a complete set of stimulus parameters, such as spatial and temporal frequency, luminance, and others. Although numerous contrast sensitivity measurements can be found in the literature, none fully explains the complete space of stimulus parameters. Therefore, in this work, we first collect and consolidate contrast sensitivity measurements from 18 studies, which explain the sensitivity variation across the parameters of interest. Then, we build an analytical contrast sensitivity model that explains the data from all those studies. The proposed castleCSF model explains the sensitivity as the function of spatial and temporal frequencies, an arbitrary contrast modulation direction in the color space, mean luminance, and chromaticity of the background, eccentricity, and stimulus area. The proposed model uses the same set of parameters to explain the data from 18 studies with an error of 3.59 dB. The consolidated contrast sensitivity data and the code for the model are publicly available at https://github.com/gfxdisp/castleCSF/.

## Introduction

A contrast sensitivity function (CSF) is a visual model that explains what is the smallest contrast of a simple stimulus (a sinusoidal grating, a Gabor patch, or a disk) that can be detected on a uniform background (Barten, 1999; Kelly, 1979; Robson, 1966). These types of detection characteristics are fundamental performance measures of the visual system and have been thoroughly measured over several decades using psychophysical methods. Despite the abundance of data, few models summarize these measurements across more than three of the many parameters influencing the visibility of the stimuli. Creating such a model is the goal of our work. Our model explains the available psychophysical data across the most relevant stimulus parameters: the chromaticity of the background, the chromatic direction of modulation, area, spatial and temporal frequency, luminance, and eccentricity. The first letters of those dimensions form the acronym of our model—castleCSF. To facilitate multiple applications and research on sensitivity, we made our model and the data available at https://github.com/gfxdisp/castleCSF.

Although contrast sensitivity explains visual performance only for a limited class of artificial stimuli, it is a building block of more complex models of the visual system, which can generalize to more comprehensive stimuli, including images or videos. For example, whereas contrast masking has different characteristics depending on the luminance, spatial frequency, and color modulation direction of the patterns, masking characteristics can be unified when contrast is normalized by the sensitivity predicted by the CSF (Cass, Clifford, Alais, & Spehar, 2009; Daly, 1992). Although the perceived magnitude of suprathreshold contrast varies across luminance, the deviation from contrast constancy can once again be explained using contrast sensitivity data (Kulikowski, 1976; Peli, 1995). The detection of more complex patterns can be estimated using energy models, which also rely on the CSF (Watson, 2000). Finally, visible differences on complex backgrounds can be explained by multichannel visual difference predictors (Daly, 1992; Mantiuk, Kim, Rempel, & Heidrich, 2011), which incorporate models of both visual masking and contrast sensitivity.

CSFs are also commonly used to control and optimize display applications, and in particular virtual and augmented reality (VR/AR) headmounted systems. In this work, we focus on the dimensions of the CSF that are the most relevant in the context of color video applications, in particular for AR/VR. Notably, as these displays typically cover a significantly larger field-of-view than traditional displays, much of the content lies outside the user’s fovea, and despite presenting very high physical pixel density and resolution, the effective pixel per visual degree resolution is often lower than conventional displays. Perceptually informed rendering algorithms, like contrast-aware (Tursun et al., 2019) or attention-aware (Krajancich, Kellnhofer, & Wetzstein, 2023) foveated rendering, and foveated image reconstruction (Kaplanyan et al., 2019) are proposed to achieve high perceptual content quality while conforming to the limited power and compute budget. Owing to their head-tracked nature, AR/VR often requires ultra-high refresh rates and low persistence values, resulting in heavier computational and bandwidth costs. Rendering modalities where a subset of frames are simplified can be used to alleviate this issue (Denes, Maruszczyk, Ash, & Mantiuk, 2019). Recent work by Duinkharjav et al. (2022) explicitly models foveated color discrimination to generate chromatic distortions that can minimize display power usage. A comprehensive model of chroma-aware contrast sensitivity is especially important to guide these kinds of AR/VR algorithm development and is provided by castleCSF.

### Factors affecting contrast sensitivity

CSFs describe the visibility of gratings through Fourier analysis (Campbell & Robson, 1968)—explaining the detection characteristic as a function of spatial and temporal frequency of the visual stimulus. This is motivated by the existence of visual channels, which are tuned to bands of spatial and temporal frequencies (Kulikowski & Robson, 1999). However, other parameters of the stimulus have no lesser influence on the sensitivity. The sensitivity is affected by luminance and chromaticity of the background (Blackwell, 1946; Xu, Ye, Mantiuk, & Luo, 2022), the size of the stimulus (Rovamo, Luntinen, & Näsänen, 1993), the angle from the fixation point (eccentricity) (Virsu & Rovamo, 1979; Wright & Johnston, 1983) and the position in the peripheral visual field (nasal, temporal, superior, or inferior) (Anderson, Mullen, & Hess, 1991), the orientation of a grating (Campbell, Kulikowski, & Levinson, 1966), and the direction along which the contrast is modulated in a three-dimensional color space (Mullen, 1985; Wuerger et al., 2020). It is also recognized that the sensitivity decreases with age, mostly owing to optical factors, such as the increase in the wavelength-dependent opacity (Pokorny, Smith, & Lutze, 1987) or the inability of the pupil to dilate at low light (senile miosis) (Watson & Yellott, 2012), but also owing to neural factors. The sensitivity is reduced at shorter viewing distances owing to the error in accommodation (Hernández, Doménech, Seguí, & Illueca, 1996). When a field of different luminance surrounds a detected pattern, the sensitivity is reduced (Yi et al., 2022) by glare and local luminance adaptation (Vangorp, Myszkowski, Graf, & Mantiuk, 2015). Binocular sensitivity is higher than monocular sensitivity and can be predicted from monocular sensitivity by quadratic summation (S binocular =SL2+SR2) (Legge, 1984).

### Measurements of contrast sensitivity

Measurements are most often conducted via noninvasive psychophysical methods (Campbell & Green, 1965; Pointer & Hess, 1989), where participants are shown stimuli of different contrast, and their performance in a detection task is measured. Alternative approaches use electrophysiological methods such as electroretinography (Hood & Birch, 1990) and visual evoked potential recordings (Norcia, Tyler, & Hamer, 1990), where the electrical response to the stimulus that originates in the retina or the visual cortex is measured directly. The characterization of optical characteristics using techniques such as optical coherence tomography (Adam, Shrier, Ding, Glazman, & Bodis-Wollner, 2013) and retinal imaging (Ortiz, Jiménez, Pérez-Ocón, Castro, & González-Anera, 2010) can also provide insights into contrast sensitivity. Data from all these different kinds of studies is often impossible to compare directly owing to the differences in methodology and control conditions. Even among psychophysical studies, there is no clear consensus regarding standard test conditions or the type of stimuli used to measure the visual system’s responses.

Because of the very large space of possible stimulus parameters, existing contrast sensitivity studies typically measure the variation along two to three selected parameters to maintain a feasible length of a study. It follows that creating a CSF that models a larger set of dimensions requires combining data from multiple studies. This poses several challenges. First, each measurement often uses slightly different stimuli (e.g., Gabors, stimuli with sharp edges, annulus rings, different shapes of the aperture), as well as different protocols and viewing conditions (monocular vs. binocular, natural vs. artificial pupil). To account for these variations, we allow for small relative changes in the sensitivity between the datasets. Moreover, many dimensions of the modeled CSF show strong interactions. For example, the spatial and temporal frequency dimensions of the CSF are not separable and cannot be modeled as a combination of independent spatial and temporal CSFs. To create a comprehensive model, it is necessary to fit a model to multiple datasets at the same time. This approach is different from what is found in previous work modeling multidimensional CSFs (Barten, 1999; Watson & Ahumada, 2016), in which datasets were fitted one at a time. For the sake of simplicity, castleCSF also does not attempt to faithfully reproduce early vision mechanisms, such as those modeled in the ISETBIO package (Brainard et al., 2015). We intend to create a practical model, which can summarize and predict a large body of data from the literature.

### Modeling contrast sensitivity

Several relevant CSF models are listed in Table 1 and are discussed in this section. Kelly was one of the first to introduce a model of the interaction of spatiotemporal mechanisms (Kelly, 1979). Notably, Kelly pointed out that the spatiotemporal CSF can alternatively be represented as the function of spatial frequency and retinal velocity, which can be more relevant for some applications. His model was based on measurements with retinal images stabilized via eye tracking, which is not representative of typical image or video content consumption use cases. We found Kelly’s data to be too distinct from comparable measurements with non-stabilized stimuli to be used in our model. Daly (2001) extended Kelly’s model, which was later fitted to data for non-stabilized stimuli (Laird, Rosen, Pelz, Montag, & Daly, 2006). All these models factor only achromatic stimuli and do not consider other stimulus properties such as luminance or stimulus area.

**Table 1.** Selection of popular CSF models from the literature. Notes: (1) for stabilized retinal images. (2) Only modeled for the three selected color directions. (3) Excluding the region in which spatial frequency is below about 10 cpd, and temporal frequency is below 10 Hz.

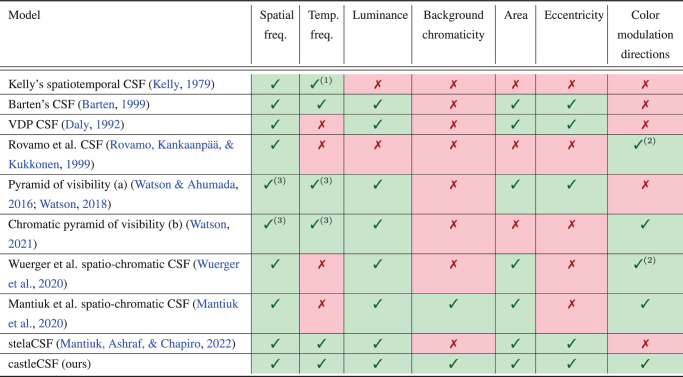

A comprehensive CSF model can be found in the visual difference predictor (VDP) work of Daly (1992), though details on how the model was fitted to data are omitted. This method models the effect of spatial frequency, luminance, area, and eccentricity on the contrast sensitivity of static achromatic stimuli. (Barten, 1999), proposed a comprehensive CSF, explicitly modeling the optical transfer function, photoreceptor and neural noise, and lateral inhibition. The original model included the effect of spatial frequency, area, and luminance (Barten, 1992). The full model also includes extensions to the parafoveal and temporal domain (Barten, 1999). Moreover, Bozorgian, Pedersen, & Thomas (2022) have shown that Barten’s model could be modified to improve predictions for peripheral contrast sensitivity and validated this with a dataset from (Thibos, Still, & Bradley, 1996). Barten’s model parameters were fitted individually to multiple datasets from the literature, demonstrating its ability to explain a wide range of measurements. Despite this, in our experiments with Barten’s CSF, we found it unable to explain more recent datasets measured at high luminance levels (Wuerger et al., 2020), in particular, the loss of sensitivity at high luminance for low-frequency achromatic Gabor patterns. This model also lacks the chromatic component necessary to represent sensitivity to color modulations.

One of the few works that model chromatic stimuli was proposed by (Rovamo, Kankaanpää, & Kukkonen, 1999). The authors modeled the low-pass behavior of spatial CSFs for isoluminant stimuli, as these do not generate lateral inhibition. The detection mechanism was modeled as the combination of optical and neural modulation transfer functions, both affected by noise. The model explained the physiological mechanisms that lead to differences between the detection of achromatic and chromatic gratings and produced good fits to Mullen’s contrast sensitivity data for isoluminant stimuli (Mullen, 1985). However, the model cannot be easily generalized to an arbitrary color modulation direction and, therefore, cannot be used to predict data from many of our datasets. In addition to these chromatic CSF models, some recent studies suggested that spatiochromatic CSFs can be inferred from the features of deep neural networks trained on low- and middle-levels tasks, such as image-denoising, autoencoding, edge detection and object recognition (Akbarinia, Morgenstern, & Gegenfurtner, 2023; Li, Gomez-Villa, Bertalmío, & Malo, 2022). To demonstrate that, the authors train a classifier to discriminate contrast and use it as a decision criterion in a two-alternative-choice contrast detection experiment. The results, however, show only a correlation with human data and cannot be used to give accurate predictions of human sensitivity.

The pyramid of visibility is a popular simplified model of contrast sensitivity. The original model (Watson & Ahumada, 2016) accounts for spatial and temporal frequencies, and background luminance, and the extended version (Watson, 2018) adds stimulus area and retinal eccentricity parameters. The pyramid of visibility establishes linear and log-linear relationships between contrast sensitivity and the studied parameters of the stimulus. This linear behavior of the CSF is only observed at high spatial and temporal frequencies (above approximately 10 cpd or 10 Hz). A chromatic extension of the pyramid of visibility, which models the effect of spatial frequency, temporal frequency, and luminance for stimuli with modulations in both achromatic and chromatic space, has also been proposed (Watson, 2021). This model works by estimating the projections of the stimulus contrast modulation for hypothetical achromatic, red-green, and yellow-violet opponent mechanisms of the visual system. The sensitivity of each of these three mechanisms is summed together to predict the overall sensitivity for a given stimulus.

castleCSF builds and expands upon our previous models of contrast sensitivity. Wuerger et al. (2020) presented a new high-dynamic-range CSF dataset, and a CSF modeled as a function of spatial frequency, mean luminance, and stimulus size for the three cardinal chromatic directions of the visual system. (Mantiuk et al., 2020) extended this work by proposing two spatiochromatic CSFs that could additionally account for the chromaticity of the background, and any modulation direction in a color space. The model was fitted to the combined data from five spatiochromatic datasets. In another work, we proposed stelaCSF (Mantiuk, Ashraf, & Chapiro, 2022), which was limited to achromatic contrast sensitivity but introduced temporal frequency, and eccentricity. stelaCSF was fitted to 11 datasets from the literature. This work combines the models and approaches used in stelaCSF Mantiuk et al. (2022) and the postreceptoral spatiochromatic CSF (Mantiuk et al., 2020). Although Mantiuk et al. (2020) showed slightly better predictions for the cone-contrast variant of the model, we selected the postreceptoral variant as it let us better isolate achromatic and chromatic mechanisms. We use a data-driven, physiologically inspired approach to model contrast sensitivity as a function of six different stimulus properties, which is the superset of all parameters of the previous models (Table 1). We do not model the effect of orientation and the effect of the peripheral visual field (nasal, temporal, superior, or inferior) because of the lack of available datasets that are compatible with the datasets included in this study.

## Combined contrast sensitivity dataset

Our first goal was to create a comprehensive spatiotemporal chromatic contrast sensitivity model that accounts for the parameters of the stimuli (Gabors), which are the most relevant for AR/VR applications: spatial and temporal frequency, background luminance and chromaticity, the direction of contrast modulation in a color space, area, and eccentricity. As described in *“Measurements of contrast sensitivity,”* gathering a single dataset that covers all these variables is impractical, so instead we opt to combine datasets from multiple sources.

Contrast sensitivity has been studied extensively over many decades, and almost every aspect of contrast detection has been measured. The main problem is that all those measurements were done independently, each measuring a different slice of a multidimensional space that defines the detection stimuli. Our goal is to select a set of measurements that covers all stimuli dimensions of interest, standardize the representation (e.g., use the same contrast definition and color space), and gather in a format that could be used to fit a single model that explains all the data.

### Contrast units and color space

As the measure of sensitivity, we use the inverse of cone contrast, where cone contrast is expressed as:

(1) $$\begin{array}{c} C\;=\;\frac{1}{\sqrt{3}}\sqrt{{\left(\frac{\Delta L}{L_{0}}\right)}^{2}+{\left(\frac{\Delta M}{M_{0}}\right)}^{2}+{\left(\frac{\Delta S}{S_{0}}\right)}^{2}},\; \end{array}$$

where ΔL, ΔM, and ΔS are the differential cone responses for the stimuli and $L_{0}$, $M_{0}$, and $S_{0}$ are the cone responses for the corresponding background. L, M, and S cone responses are given by

(2) $$\begin{array}{c} L\;=\;0.689903\int_{\lambda }l_{2}\left(\lambda \right)\;E\left(\lambda \right)\;d\lambda ,\; \end{array}$$

(3) $$\begin{array}{c} M\;=\;0.348322\int_{\lambda }m_{2}\left(\lambda \right)\;E\left(\lambda \right)\;d\lambda ,\; \end{array}$$

(4) $$\begin{array}{c} S\;=\;0.0371597\int_{\lambda }s_{2}\left(\lambda \right)\;E\left(\lambda \right)\;d\lambda ,\; \end{array}$$

where *l*_2_, *m*_2_, and *s*_2_ are 2° CIE 2006 cone fundamentals (CIE, 2006)^1^ and *E* is the measured spectral radiance emitted from the display.

Most achromatic contrast sensitivity studies report background luminance (or retinal illuminance) but do not report the spectral composition or chromaticity of the background. For those, we assumed L, M, and S cone responses that corresponded to the D65 illuminant and the modulation along the first (L+M) dimension in the DKL color space (Derrington, Krauskopf, & Lennie, 1984). For chromatic datasets, we either received the spectral emission characteristic from the authors or assumed typical emission spectra of the display used in the study (e.g., emission spectrum of a CRT monitor).

A common practice when measuring chromatic contrast sensitivity is to isolate the chromatic mechanisms of each individual using a heterochromatic flicker paradigm (Wagner & Boynton, 1972). Since our goal is to create a model of an average observer, which is suitable for general applications, we excluded the datasets that measured different color modulation directions for each observer.

### Datasets

We selected 18 datasets to train and test our model. Table 2 lists nine datasets with only achromatic stimuli and the achromatic portions of four datasets that contained both achromatic and chromatic stimuli. Table 3 lists five chromatic datasets, along with the chromatic portions of the four mixed datasets. Notably, Table 2 contains all the datasets used in recent work by Mantiuk et al. (2022), along with the recently collected HDR disc CSF dataset by Ashraf, Mantiuk, & Chapiro (2023), but excluding the data from Anderson et al. (1991). We excluded that dataset because it used a different detection criterion than all other datasets (discrimination rather than detection) and its measurements were not comparable. We also avoided the datasets that were collected for an artificial pupil, because we are interested in visual performance for natural viewing. If multiple datasets in the literature covered a similar range of contrast sensitivity parameters, we selected those that were collected for a larger and more representative group of observers.

**Table 2.** The achromatic datasets used in our study. Asterisks (*) mark cases where the data were obtained directly from the authors. In the remaining cases, the data were scanned from plots in the relevant publication. All listed datasets measured contrast sensitivity using contrast detection tasks. For datasets with more than one observer where individual data were provided, the expected interobserver variability (standard deviation) within each condition in dB is shown along with the number of observers.

Dataset Name	Spatial freq. cpd	Temporal freq. Hzg	Luminance cd/m^2^	Eccentricity deg (visual field)	Area deg^2^	Stimulus	Interobserver variability dB
Robson, 1966	0.5 – 30	0.5 – 32	20	0	6.25	Grating with rectangular aperture	✗
Virsu & Rovamo, 1979	0.5 – 16	✗	10	0, 5, 10, 15, 20, 25, 30 (nasal)	19.65	Grating with circular aperture	✗
Virsu, Rovamo, & Laurinen, 1982	1 – 22.6	1, 18	10	0, 1.5, 4, 7.5, 14, 30 (nasal)	1.57	Grating with semi-circular aperture	✗
Wright & Johnston, 1983	0.25, 2, 6, 9	0, 0.25, 8, 16	100	0 – 12 (superior)	0.75, 2.35, 36	Grating with rectangular aperture	0.4748 (*N* = 2)
Rovamo et al., 1993	0.125, 0.25, 0.5, 1, 2, 4, 8, 16, 32	✗	50	0	0.003 – 980	Grating with rectangular aperture	✗
Snowden, Hess, & Waugh, 1995	0.25, 1, 2, 4, 5, 10, 20	0.8 – 55.7	0.02 – 870	0	0.25, 1, 4.01	Gabor patch	✗
Modelfest (Watson, 2000) (*)	1.12 – 30	✗	30	0	0.003 – 0.78	Gabor patch	3.7464 (*N* = 16)
Colorfest (Wuerger, Watson, & Ahumada, 2002) (*)	1.2 – 30	✗	40	0	0.78	Gabor patch	2.6553 (*N* = 3)
Laird et al., 2006	4, 8, 16	9.2 – 31.4	60	0	4.75	Gabor patch	✗
Hansen, Pracejus, & Gegenfurtner, 2009	✗	✗	100	10, 20, 30, 40, 50	50.27	Discs	✗
HDR-VDP CSF (Kim, Mantiuk, & Lee, 2013; Mantiuk et al., 2011) (*)	0.125 – 32	✗	0.002, 0.02, 0.2, 2, 20, 150	0	0.07, 0.78, 7.06	Fixed cycles Gabor patch	2.7233 (*N* = 10)
HDR CSF (Wuerger et al., 2020) (*)	0.5, 1, 2, 4, 6, 12, 24	✗	0.02, 0.2, 2, 20, 200, 2000, 7000, 10000	0	0.005 – 12.5	Fixed cycles Gabor patch	2.7873 (*N* = 23)
HDR disc CSF (Ashraf et al., 2023)	✗	✗	0.02, 0.2, 2, 20, 200	0	0.02, 0.2, 3.14	Discs	2.4710 (*N* = 12)

**Table 3.** The chromatic datasets used in our study. Asterisks (*) mark cases where the data were obtained directly from the authors. In the remaining cases, the data were scanned from plots in the relevant publication. All listed datasets measured contrast sensitivity using contrast detection tasks. For datasets with more than one observer where individual data were provided, the mean interobserver variability in dB (standard deviation within each condition) is shown along with the number of observers.

Dataset Name	Chromatic modulation	Spatial freq. cpd	Temporal freq. Hz	Luminance cd/m^2^	Eccentricity deg (visual field)	Area deg^2^	Stimulus	Inter-observer variability dB
Van der Horst & Bouman, 1969	Yellow-blue and red-bluish green over grey (equal-energy point) background, Red-green over yellow background	0.7 – 18	0, 0.5 – 9.5	0.009 – 70	0	14.84	Grating with rectangular aperture	✗
Colorfest (Wuerger et al., 2002) (*)	Green-red, yellow green-violet, Greenish-pink, yellow-blue, dark green-light pink, dark yellow-light blue over D65 background	1.2 – 30	✗	40	0	0.78	Gabor patch	4.1975 (*N* = 3)
Hansen et al., 2009	Pinkish red and violet over grey background	✗	✗	100	10, 20, 30, 40, 50	50.27	Discs	✗
Kim et al., 2013	Green-red, yellow green-violet, dark green- light pink, dark yellow-light blue over D65 background	0.25, 0.5, 1, 2, 4	✗	0.02, 0.2, 2, 20, 150	0	7, 28	Gabor patch	3.1301 (*N* = 7)
Lucassen, Lambooij, Sekulovski, & Vogels, 2018	Four-color directions modulated in u’v’ color space 45° apart from each other over 3 different backgrounds (grey, light yellow and dark yellow)	0.15, 0.3, 0.15, 1.5, 3, 5	✗	108	0	280	Gabor patch	✗
Kong, Pérez, Vogels, Sekulovski, & Heynderickx, 2018 (*)	0°, 45°, 90°, 135° in u’v’ color space over 9 different backgrounds	✗	2, 4, 8, 10, 15, 20, 25	35	0	78.5	Discs	6.6586 (*N* = 3)
HDR CSF (Wuerger et al., 2020) (*)	Red-green and yellow-violet over D65 background	0.125, 0.25, 0.5, 1, 2, 4, 6, 12, 24	✗	0.02, 0.2, 2, 20, 200, 2000, 7000, 10000	0	0.005 – 50	Fixed cycles Gabor patch	3.0668 (*N* = 23)
HDR disc CSF (Ashraf et al., 2023) (*)	Pinkish red and violet over D65 background	✗	✗	0.02, 0.2, 2, 20, 200	0	0.02, 0.2, 3.14	Discs	4.2842 (*N* = 10)
Five Centers (Xu, Luo, & Sekulovski, 2020) (*)	Six-color directions over red, white, cyan, blue and yellow backgrounds	0.06, 0.12, 0.24, 0.48, 0.96, 1.92, 3.84	✗	8.8, 14.1, 24, 50, 72	0	272	Gabor patches	✗

### Aperture

The type of stimulus used for each dataset is listed in the last column of each table (Tables 2 and 3). Most datasets measured contrast thresholds using Gabor patches. Conversely, some datasets used sine gratings with rectangular or circular apertures instead of smooth Gaussian windows. To standardize these modalities, given a circular aperture with a diameter *d*, we assumed the stimulus to be equivalent to a Gabor patch with $\sigma =\frac{d}{2}$. For rectangular apertures with the given area *a*, we assume the stimuli to be equivalent to a disc aperture with the same area (πσ^2^ = *a*). Although those relationships may not fully explain the differences between Gaussian and other apertures, any inaccuracies are compensated for when fitting and allowing for a sensitivity shift in each dataset (see Eq. (32)).

Datasets that were directly obtained from the authors are marked with asterisks. The remaining datasets were scanned from the corresponding papers using the WebPlotDigitizer tool (Rohatgi, 2022). To ensure this scanning method had sufficiently high accuracy and repeatability we performed an experiment where a plot for which the ground truth data was available (achromatic CSF at 200 cd/m^2^ from Figure 5 in Wuerger et al., 2020) was scanned five times. The mean standard deviation of the log sensitivity values was found to be very low (0.00534 dB). The root mean squared error (RMSE) error between the mean of the 5 scans and the ground truth data was also modest at 0.0117 dB, or 0.135% of the original data. This leads us to conclude that the scanning tool offers a robust method for gathering data.

**Figure 1.**
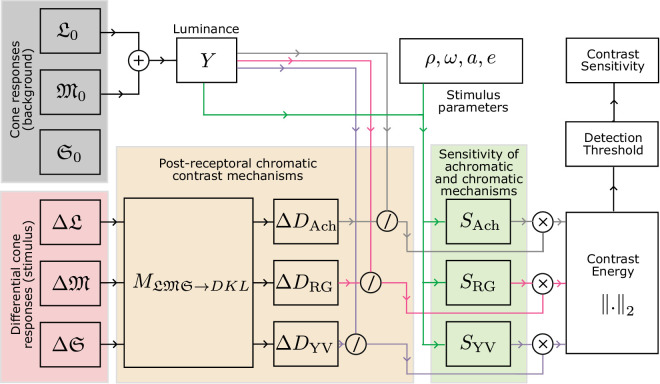
Contrast encoding and the main processing stages of castleCSF. The color of the background is represented as cone responses ($L_{0}$, $M_{0}$, $S_{0}$) and the direction in the color space as the increments of cone responses (ΔL, ΔM, ΔS). The direction is transformed into the DKL color space, and the contrast is computed by dividing by luminance, where luminance is the sum of $L_{0}$ and $M_{0}$. The contrast for the three cardinal color directions is then modulated by the sensitivity of each mechanism (*S*_Ach_, *S*_RG_, and *S*_YV_) and pooled to obtain the contrast energy. We use this energy to find the detection threshold, and finally compute the sensitivity value.

**Figure 2.**
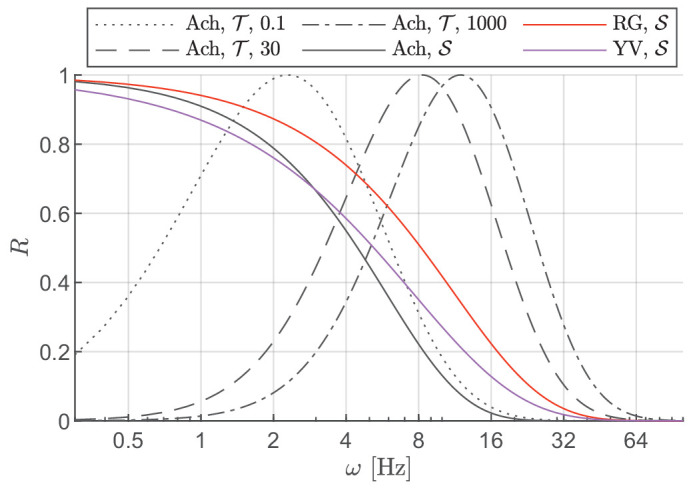
Responses of the temporal channels. The three sustained (S) channels are low-pass, while the achromatic transient (T) channel is bandpass. The response of this bandpass channel depends on luminance—shifts towards higher frequencies as the mean luminance increases.

**Figure 3.**
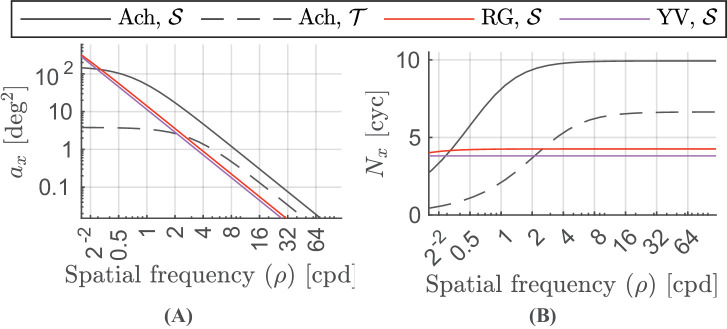
(**A**) Change in the critical area with spatial frequency. The critical area parameter for the achromatic sustained channel saturates at about 0.5 cpd and for the achromatic transient channel saturates at about 2 cpd. For the red-green and yellow-violet channels, this saturation point lies at a much smaller spatial frequency. (**B**) Critical number of cycles changing with the spatial frequency. The value of the critical number of cycles becomes constant with increasing spatial frequency. For the red-green and yellow-violet channels, our model predicts the critical number of cycles to be constant for approximately the whole range of spatial frequencies detectable by the human visual system.

**Figure 4.**
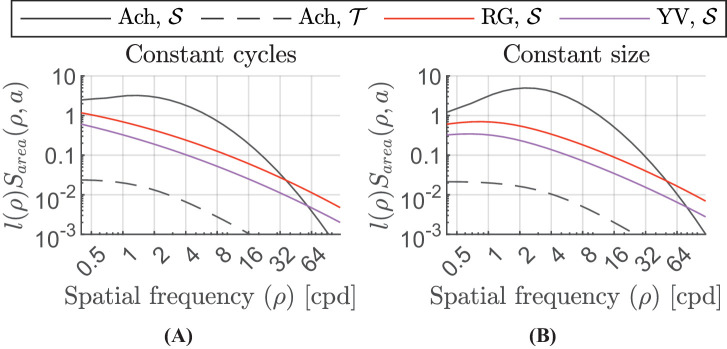
Joint effect of spatial frequency and area on sensitivity. (**A**) Contrast sensitivity of stimuli with four visible cycles at each spatial frequency. (**B**) Contrast sensitivity of stimuli with a constant radius of 2° at each spatial frequency.

**Figure 5.**
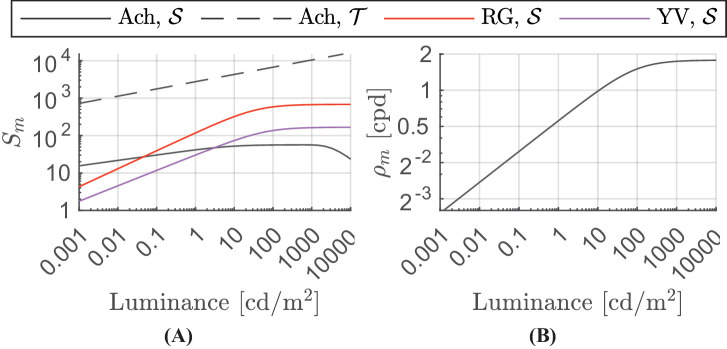
(**A**) Luminance-dependent shift in sensitivity. The responses of the three sustained channels increase with luminance and then saturate, with the achromatic sustained channel response showing a decline at very high luminances. The transient channel response shows a linear relationship with luminance in log-log space. (**B**) Luminance-dependent spatial frequency shift for the achromatic sustained channel. The value of the ρ_m_  parameter for the remaining channels is independent of luminance.

## Contrast sensitivity function (CSF)

In this section, we explain how the proposed castleCSF model can predict the detection threshold for any background color, specified in cone response units ($L_{0}$, $M_{0}$, $S_{0}$), and for any color modulation direction, specified as increments of cone responses (ΔL, ΔM, ΔS). A high-level diagram and description of the model’s workflow is shown in Figure 1.

To isolate the three color mechanisms, one achromatic and two chromatic, we transform the increments to the color-opponent DKL space (Derrington et al., 1984) assuming a D65 grey background using the transformation matrix:

(5) $$\begin{array}{c} \;\left[\begin{array}{c} \Delta D_{\text{Ach}}\\ \Delta D_{\text{RG}}\\ \Delta D_{\text{YV}} \end{array}\right]\;=\;\left[\begin{array}{ccc} 1 & 1 & 0\\ 1 & -2.3112 & 0\\ -1 & -1 & 50.9875 \end{array}\right]\cdot \left[\begin{array}{c} \Delta L\\ \Delta M\\ \Delta S \end{array}\right].\; \end{array}$$

To compute contrast, we divide the color-opponent increments by the background luminance:

(6) $$\begin{array}{c} \;\Delta C_{\text{Ach}}=\frac{\Delta D_{\text{Ach}}}{Y},\;\;\Delta C_{\text{RG}}=\frac{\Delta D_{\text{RG}}}{Y},\;\;\Delta C_{\text{YV}}=\frac{\Delta D_{\text{YV}}}{Y},\;\;\;\;\; \end{array}$$

where $Y=L_{0}+M_{0}$. The contrast values of the three mechanisms are weighted by the sensitivity functions *S*_Ach_, *S*_RG_, and *S*_YV_, and pooled together into a contrast energy:

(7) $$E=\sqrt{\sum_{c\in \{\text{Ach},\text{RG},\text{YV}\}}{\left(S_{c}\left(\rho ,\omega ,Y,a,e\right)\;\Delta C_{c}\right)}^{2}},\;$$

*S*_*c*_( · ) are the sensitivity functions of each mechanism, which we describe in the next section.

We assume that the contrast is detected when the contrast energy *E* = 1. Such a threshold can be found analytically by introducing a constant *t*:

(8) $$\begin{array}{c} \;\Delta C_{\text{Ach}}=t\;\Delta {\hat{C}}_{\text{Ach}},\;\Delta C_{\text{RG}}=t\;\Delta {\hat{C}}_{\text{RG}},\;\Delta C_{\text{YV}}=t\;\Delta {\hat{C}}_{\text{YV}},\;\;\;\;\;\;\; \end{array}$$

where $\left(\Delta {\hat{C}}_{\text{Ach}},\Delta {\hat{C}}_{\text{RG}},\Delta {\hat{C}}_{\text{YV}}\right)$ is the contrast at the detection threshold. If we introduce these expressions to Eq. (7), we get:

(9) $$\;\;\;\;\;\;\;\;\;\;\;\;\;\;E=t\sqrt{\sum_{c\in \{\text{Ach},\text{RG},\text{YV}\}}{\left(S_{c}\left(\rho ,\omega ,Y,a,e\right)\;\Delta {\hat{C}}_{c}\right)}^{2}}\;=t\;\hat{E},$$

where $\hat{E}$ is the contrast energy at the threshold. Since we assumed that the contrast energy should be 1 at the detection threshold, we have $\hat{E}=1$ and hence *t* = *E*. This shows us that the detection threshold expressed as an increment in the color opponent space can be computed from Eq. (8) as:

(10) $$\begin{array}{c} \;\Delta {\hat{C}}_{\text{Ach}}=\frac{\Delta C_{\text{Ach}}}{E},\;\Delta {\hat{C}}_{\text{RG}}=\frac{\Delta C_{\text{RG}}}{E},\;\Delta {\hat{C}}_{\text{YV}}=\frac{\Delta C_{\text{YV}}}{E}.\;\; \end{array}$$

Assuming a local linearity of the transformation around the background color ($L_{0}$, $M_{0}$, $S_{0}$), the same approximately holds for the increment thresholds in the cone response space:

(11) $$\begin{array}{c} \Delta \hat{L}\;\approx \;\frac{\Delta L}{E},\;\Delta \hat{M}\;\approx \;\frac{\Delta M}{E},\;\Delta \hat{S}\;\approx \;\frac{\Delta S}{E}.\; \end{array}$$

Once we know the cone response increments at the threshold, we can calculate the sensitivity as the inverse of the cone contrast from Eq. (1):

(12) $$\begin{array}{c} S\;=\;\sqrt{\frac{3}{{\left(\frac{\Delta L}{L_{0}\;E}\right)}^{2}+{\left(\frac{\Delta M}{M_{0}\;E}\right)}^{2}+{\left(\frac{\Delta S}{S_{0}\;E}\right)}^{2}}},\; \end{array}$$

where *E* is the energy from Eq. (7).

In the following sections, we explain how the sensitivities of the three mechanisms, *S*_Ach_( · ), *S*_RG_( · ), and *S*_YV_( · ), are modeled.

### Mechanism sensitivity

We model the sensitivity of each mechanism, *S*_Ach_( · ), *S*_RG_( · ) and *S*_YV_( · ), by considering the effects of spatial frequency, temporal frequency, luminance, area, and eccentricity. The following sections explain how each factor is modeled.

#### Temporal frequency

A series of early work by de Lange introduced the concept of distinct temporal channels in the human visual system by reporting varying integration time constants for achromatic and chromatic stimuli at different flicker rates (de Lange, 1958a; de Lange, 1958b). The site of these mechanisms (retinal, LGN, cortical, etc.), their inputs (from parvo or magnocellular pathways), and the number of these temporal channels is still the subject of an ongoing debate. Results from psychophysical studies have suggested the presence of multiple temporally tuned channels (Hess & Snowden, 1992; Kelly, 1983; Metha & Mullen, 1996; Robson, 1966). For near-threshold achromatic stimuli, three temporal channels have been proposed with two motion and one flicker detection channel (King-Smith & Kulikowski, 1975; Mandler & Makous, 1984). We opted for the two-channel (low-pass/sustained and band-pass/transient) model supported by studies such as Tolhurst (1973) and Anderson and Burr (1985) for the achromatic mechanism because of its relative simplicity, and because the potential third high temporal frequency channel likely only detects low spatial frequencies (Hess & Snowden, 1992). Some temporal CSF data suggest that the peak of the achromatic transient channel dependent on luminance (de Lange, 1958a; Snowden et al., 1995), and shifts toward lower temporal frequencies as the luminance level decreases. For chromatic mechanisms, there is evidence of one slow sustained chromatic channel and a faster transient channel (Cass et al., 2009; Cropper & Wuerger, 2005; Gegenfurtner & Hawken, 1996; Metha & Mullen, 1996). We chose not to include the chromatic transient channels in our model because their magnitudes are relatively smaller than the chromatic sustained channels (Cass et al., 2009), and thus contribute little to the overall sensitivity response. It has also been suggested that the higher (temporal) frequency chromatic stimuli might be mediated by luminance mechanisms instead of a dedicated chromatic transient channel (Dobkins, Gunther, & Peterzell, 2000).

The impulse responses of the temporal filters associated with the sustained (S) and transient (T) channels can be well approximated by the generalized exponential functions (Mantiuk et al., 2022):

(13) $$\begin{array}{c} \;R_{S}^{c}\left(\omega \right)\;=\;\exp\left(-\frac{\omega^{\beta_{S}^{c}}}{\sigma_{S}^{c}}\right),\;\forall c\in \left\{\text{Ach},\text{RG},\text{YV}\right\},\; \end{array}$$

and:

(14) $$\begin{array}{c} \;R_{T}^{\mathrm{Ach}}\left(\omega ,Y\right)=\exp\left(-\frac{{\left|\omega^{\beta_{T}^{\mathrm{Ach}}}-{\left(\omega_{0}\left(Y\right)\right)}^{\beta_{T}^{\mathrm{Ach}}}\right|}^{2}}{\sigma_{T}^{\mathrm{Ach}}}\right),\; \end{array}$$

where ω is the temporal frequency in Hz, and $\beta_{S}^{c}$, $\sigma_{S}^{c}$, βT Ach , and σT Ach  are the parameters of the model. ω_0_  is the peak temporal frequency of the transient channel which was fixed at 5 Hz in stelaCSF (Mantiuk et al., 2022). In castleCSF, we model this as a parameter dependent on the mean luminance level based on data from de Lange (1958a) and Snowden et al. (1995). We found that the following linear relationship between the transient channel peak frequency and log luminance level explained the data well:

(15) $$\begin{array}{c} \omega_{0}\left(Y\right)\;=\;m_{\omega }^{\mathrm{Ach}}\log_{10}Y+c_{\omega }^{\mathrm{Ach}}\;\left[\mathrm{Hz}\right],\; \end{array}$$

where mω Ach  and cω Ach  are the fitted parameters. This assumption that, at higher luminance levels, the visual system is more attuned to higher temporal frequencies is also consistent with the information flow theory of early vision proposed by Van Hateren (1993) where they showed that the signal-to-noise ratio increases as a function of log light intensity and that the peak of the neural filter (analogous to the transient channel in our model) shifts towards higher frequencies with increasing signal-to-noise ratio (SNR). Ferry-Porter’s law, which states that the critical flicker fusion frequency (CFF, the temporal frequency at which flicker can no longer be perceived) increases linearly with the logarithm of the luminance level (Ferry, 1892; Porter, 1902), also supports our model because increasing peak temporal frequency would also result in a higher cut-off frequency or CFF. The original works by Ferry and Porter do not suggest any saturation point for luminance level beyond which the CFF would no longer increase but the series of studies by Hecht et al. (Hecht & Verrijp, 1933; Hecht & Shlaer, 1936; Hecht & Smith, 1936) and more recently by Chapiro, Matsuda, Ashraf, and Mantiuk (2023) have shown that CFF reaches a saturation point depending on the size and eccentricity of the stimulus. This would imply a sigmoidal instead of linear relationship in Eq. (15). However, given limited data, we opted for a simpler model.

The responses of the temporal channels in our model are shown in Figure 2. All the sustained channels are low-pass, while the achromatic transient channel is band-pass with the peak shifting toward higher temporal frequencies as the luminance increases.

#### Spatial frequency

The spatial frequency response of the achromatic mechanism (*S*_Ach_ in Figure 1) is modeled to be band-pass. The assumption that the spatial frequency response for achromatic static gratings is band-pass is well-supported in visual perception literature. Studies such as those by (Blakemore & Campbell, 1969) demonstrate that human visual sensitivity is highest at intermediate spatial frequencies and falls off at both low and high spatial frequencies. The magnitude and the location of the peak sensitivity vary depending on the other properties (luminance, temporal frequency, size, etc.) of the stimulus. At the low spatial frequency end, the sensitivity fall-off is caused by lateral inhibition, but this inhibitory response is not strong when the low spatial frequency stimulus is temporally-modulated (Donner & Hemilä, 1996; Tolhurst, 1973) and results in a low-pass response. We model the spatial frequency responses of both the sustained and temporal achromatic channels as log-parabolas (Ahumada & Peterson, 1992):

(16) $$\begin{array}{c} l_{t}^{c'}\left(\rho ,Y\right)\;=\;10^{-\frac{{\left(\log_{10}\rho -\log_{10}\rho_{m,t}^{c}\left(Y\right)\right)}^{2}}{2^{k_{b,t}^{c}}}},\; \end{array}$$

where *c* ∈ {Ach, RG, YV} is the index of the mechanism, t∈{S,T} is the temporal channel (sustained or transient), $\rho_{m,t}^{c}$ controls the position of the peak of the parabola as a function of luminance (described in *Luminance*), and $k_{b,t}^{c}$ controls the bandwidth of the log-parabola. For achromatic spatial contrast sensitivity, the parabola is truncated on the low spatial frequency side of the envelope:

(17) ltAch(ρ)=1-ka,tAchifρ<ρm,tAchandltAch'<1-ka,tcltAch'(ρ)otherwise

where, t∈{S,T} and ka,t Ach  is a function parameter that controls the decrease in achromatic spatial contrast sensitivity at low frequencies. Our model fits coincide with the findings from the literature that the sustained channel has a band-pass shape with the peak shifting with different stimuli properties. The fitted value of the peak spatial frequency parameter for the transient channel (kρ,T Ach  in Table 5) has a very small value, which results in a low-pass shape.

**Table 4.** Prediction errors of contrast sensitivity models, tested on the same subset of data points. The errors are reported in the dB units (see Eq. (33)) as the mean and standard deviation across five-folds (leave-one-out cross-validation). Each column shows the result for the subset of the dataset, containing variation along selected parameters of the stimuli (selected to match the parameters supported by CSFs). The value of *N* is the number of data points included in each subset (testing and training parts). The predictions are reported for five-fold cross-validation (see text). The symbols denote: ρ, spatial frequency; *Y*, luminance; *a*, area; ω, temporal frequency; *e*, eccentricity, *c*, modulation along chromatic color directions., *c*_*DKL*_, modulation along the cardinal color direction (achromatic, red-green, yellow-violet) only. Asterisks (*) mark the cases where low spatial and temporal frequencies are removed from the datasets.

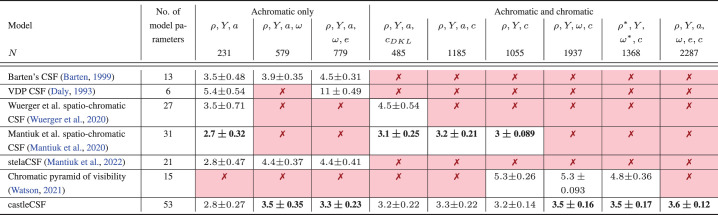

The bold text indicates the model with the minimum prediction error for that particular parameter set within the column.

The spatial frequency responses of the two chromatic mechanisms are approximately low-pass, that is, the sensitivity decreases with increasing spatial frequency. This is because of the much weaker low-spatial frequency inhibition in the chromatic detection pathways (Kelly, 1983; Metha & Mullen, 1996). The roll-off and cut-off frequency of these responses depends on the other stimulus properties. The low-pass response of the spatio-chromatic CSF can also be modeled as a truncated log-parabola $l_{t}^{c^{'}}$ with the sensitivity for lower spatial frequencies leveling off (instead of decreasing like the achromatic CSF) at the peak frequency. This function is represented as:

(18) $$\begin{array}{c} l_{S}^{c}\left(\rho \right)\;=\;\left\{\begin{array}{cc} 1 & \text{if}\;\rho <\rho_{m,S}^{c}\left(Y\right)\\ l_{S}^{c^{'}}\left(\rho \right) & \mathrm{otherwise} \end{array}\right.,\; \end{array}$$

where c∈{ RG , YV }. The definition of the parameters is the same as that for the achromatic channel.

#### Area

The stimuli become easier to detect (the sensitivity is increased) as their size increases. The contrast sensitivity increases with stimulus size for achromatic and chromatic CSFs up to a certain value, known as the critical area *a*_*x*_. The value of this critical area and the rate of increase of sensitivity up until the critical area depends on the spatial frequency of the stimuli and is modeled in (Rovamo et al., 1993) as

(19) $$\begin{array}{c} a_{x,t}^{c}\;=\;\frac{a_{0,t}^{c}}{1+{\left(\frac{\rho }{\rho_{0,t}^{c}}\right)}^{2}}\;\left[\deg^{2}\right],\; \end{array}$$

where *c* ∈ {Ach, RG, YV} and t∈{S,T}. $a_{x,t}^{c}$ is the maximum value of the critical area and $\rho_{0,t}^{c}$ is the minimum value of spatial frequency, beyond which the value of the critical area is no longer constant and begins to decrease with increasing spatial frequency, for each colour and temporal channel. Figure 3A shows the change in the critical area for the four modeled channels. The plot shows that the detection mechanism integrates over a smaller area at high frequencies. The product of a function of this critical area $a_{x,t}^{c}$ and spatial frequency is the critical number of cycles over which our visual system can integrate. Figure 3B shows the change in the critical number of cycles as a function of the spatial frequency.

The size-dependent sensitivity response follows the model of Rovamo et al. (1993) and is expressed as:

(20) $$\begin{array}{c} S_{\mathrm{area},t}^{c}\left(\rho ,a\right)\;=\;\rho \;\sqrt{\frac{a_{x,t}^{c}}{1+\frac{a_{x,t}^{c}}{a}}}\;\left[\mathrm{cyc}\right].\; \end{array}$$

The effect of size is independent of other factors in our model. The combined effect of spatial frequency and stimulus size on the contrast sensitivity is shown in Figure 4. The log-parabola bandpass shape of the achromatic sustained channel is more pronounced for the stimuli of constant size (rather than the constant number of cycles) where at low spatial frequencies fewer cycles are presented.

#### Luminance

In dim light, contrast sensitivity increases in proportion to the square root of retinal illuminance, according to the DeVries-Rose law, but in bright light contrast sensitivity follows Weber’s law and is independent of illuminance (Blackwell, 1946; Rovamo, Mustonen, & Näsänen, 1995). Kim et al. (2013) have shown similar results for chromatic stimuli, with sensitivity saturating from approximately 50 cd/m^2^. The recent sensitivity data from Wuerger et al. (2020), measured up to 10,000 cd/m^2^, shows that the assumption of sensitivity becoming constant at high luminances is not entirely accurate for achromatic stimuli as the sensitivity at very high luminance, above 1000 cd/m^2^, starts to drop, especially for low frequencies. Following Mantiuk et al. (2022), we model the change of the luminance-dependent sensitivity for achromatic sustained mechanism as an initial increase followed by a plateau and eventual decrease:

(21) $$\begin{array}{ccc} & & \;S_{m,S}^{\mathrm{Ach}}\left(Y\right)=k_{s1,S}^{\mathrm{Ach}}{\left(1+\frac{k_{s2,S}^{\mathrm{Ach}}}{Y}\right)}^{-k_{s3,S}^{\mathrm{Ach}}}{\left(1-\frac{1+k_{s4,S}^{\mathrm{Ach}}}{Y}\right)}^{-k_{s5,S}^{\mathrm{Ach}}}.\; \end{array}$$

The luminance dependence of chromatic sensitivity is modeled as an increase and then saturation of sensitivity with increasing luminance:

(22) $$\begin{array}{ccc} & & \;S_{m,S}^{c}\left(Y\right)=k_{s1,S}^{c}{\left(1+\frac{k_{s2,S}^{c}}{Y}\right)}^{k_{s3,S}^{c}},\;\;\forall c\in \left\{\text{RG},\text{YV}\right\}.\;\;\;\; \end{array}$$

For the achromatic transient channel, we again followed the approach from Mantiuk et al. (2022) and modeled the change in sensitivity as a linear function of luminance:

(23) $$\begin{array}{c} S_{m,T}^{\mathrm{Ach}}\left(Y\right)\;=\;k_{s2,T}^{\mathrm{Ach}}Y^{k_{s1,T}^{\mathrm{Ach}}}.\; \end{array}$$

This relationship is supported by the studies from Swanson, Ueno, Smith, and Pokorny (1987) and Snowden et al. (1995), showing the linear increase of sensitivity to flickering stimuli with luminance. For the luminance ranges tested in the aforementioned studies, the sensitivity did not saturate with luminance as was the case for static achromatic and chromatic stimuli.

Luminance also causes the shift of the peak sensitivity—as the luminance is decreased, the sensitivity drops but this reduction is stronger for high frequencies. These effects have different characteristics for the sustained and transient achromatic, and two chromatic mechanisms. The effect on the luminance-dependent frequency shift in the spatial log-parabola response (Eq. (16)) is modeled as:

(24) $$\begin{array}{ccc} \;\rho_{m,S}^{\mathrm{Ach}}\left(Y\right)\; & = & \;k_{\rho 1,S}^{\mathrm{Ach}}{\left(1+\frac{k_{\rho 2,S}^{\mathrm{Ach}}}{Y}\right)}^{-k_{\rho 3,S}^{\mathrm{Ach}}}\;\left[\mathrm{cpd}\right],\\ \;\rho_{m,T}^{\mathrm{Ach}}\left(Y\right)\; & = & \;k_{\rho ,T}^{\mathrm{Ach}}\;\left[\mathrm{cpd}\right],\\ \;\rho_{m,S}^{c}\left(Y\right)\; & = & \;k_{\rho ,S}^{c}\;\left[\mathrm{cpd}\right],\;\forall c\in \left\{\text{RG},\text{YV}\right\},\; \end{array}$$

where *Y* is luminance in cd/m^2^, $k_{\cdots }^{c}$ are the parameters of the model, $S_{m,S/T}^{c}$ is the luminance-dependent sensitivity, and $\rho_{m,S/T}^{c}$ is responsible for the spatial frequency shift in Eqs. (17) and (18).

We model the effect of luminance rather than retinal illuminance (in Trolands) because the former is more readily available in engineering applications, in which the pupil size (required for calculating retinal illuminance) is often unknown or difficult to measure. Figure 5 show the values of the luminance-dependent parameters in Eqs. (21) to (24). These parameters only show the effect of luminance and do not include the shift in the peak sensitivity and peak spatial frequency induced by the stimulus size. The achromatic sustained response in Figure 5 a shows the decline in sensitivity for luminances of greater than 1,000 cd/m^2^. The sensitivity of the achromatic transient channel increases with increasing luminance while both the chromatic channels transition to Weber’s region above 20 cd/m^2^. The log-parabola spatial frequency parameter for the achromatic sustained channel increases with luminance up to about 500 cd/m^2^ as shown in Figure 5B and then remains constant with further increase in luminance. An increase in this parameter translates to the shifting of the spatial contrast sensitivity curve toward higher spatial frequencies. The peak spatial frequency parameters for the achromatic transient channel and both chromatic sustained channels are constants and thus independent of luminance.

#### Eccentricity

Contrast sensitivity decreases with retinal eccentricity for achromatic (Robson & Graham, 1981) and chromatic CSFs (Anderson et al., 1991), along with other visual performance metrics such as visual acuity (Anstis, 1974), vernier acuity (Levi, Klein, & Aitsebaomo, 1985), crowding (Coates, Chin, & Chung, 2013), and so on. The performance differences between the foveal and peripheral regions of the visual field can be explained by the differences in the neural configuration of photoreceptors in different regions of the retina. The receptive field size increases with eccentricity (Hubel & Wiesel, 1962) resulting in a functional increase in spatial summation with eccentricity (Johnson, Keltner, & Balestrery, 1978; Wilson, 1970). The rate of this change depends on the color and the spatial frequency of the stimuli. If all other properties of the stimuli are kept constant, the visual system’s sensitivity to a low-frequency stimulus will decrease at a lower rate as compared to a high-frequency stimulus when the stimuli are moved from the fovea to the periphery across the retina (Virsu & Rovamo, 1979; Virsu et al., 1982). We have modeled the sensitivity drop with respect to retinal eccentricity as a log-linear function of spatial frequency and eccentricity, following (Watson, 2018):

(25) $$\begin{array}{c} S_{\mathrm{ecc}}^{c}\left(e,\rho \right)\;=\;10^{-({\hat{k}}_{e1}^{c}\;\rho \;e+{\hat{k}}_{e2}^{c}\;e)},\; \end{array}$$

where *c* ∈ {Ach, RG, YV}.

Finding a suitable peripheral chromatic contrast sensitivity dataset to validate our hypotheses proved to be challenging. The dataset from Hansen et al. (2009) primarily drove the optimization of the eccentricity-dependent parameters for the chromatic channel in our model. We did not include any datasets that used flicker photometry to isolate the chromatic mechanism as we could not parse their data in LMS cone contrasts (Anderson et al., 1991; Mullen & Kingdom, 2002; Mullen, Sakurai, & Chu, 2005; Newton & Eskew, 2003; Noorlander, Koenderink, Den Olden, & Edens, 1983). Moreover, Mullen et al. (2002) and Mullen et al. (2005) used very thin sinusoidal grating strips to avoid detection from neighboring peripheral receptive fields. The equivalent Gabor assumption (see *Aperture*) did not result in good fits for these datasets.

The decrease in sensitivity is nonuniform across the visual field, with a slower decrease in the nasal direction (Anderson et al., 1991). To model this effect, the ${\hat{k}}_{e1}^{c}$ and ${\hat{k}}_{e2}^{c}$ eccentricity response parameters from Eq. (25) are calculated as weighted means of sensitivity drop in the nasal (kei, nasal c) and other directions ($k_{ei}^{c}$).

(26) $$\begin{array}{ccc} \;{\hat{k}}_{ei}^{c}\; & = & \;\alpha k_{ei}^{c}+\left(1-\alpha \right)k_{ei,\mathrm{nasal}}^{c}\;\;\;\;\mathrm{where}\;\;i=1,2\;\;\mathrm{and}\\ \;\alpha \; & = & \;\min\left\{1,\left|\frac{\theta -180}{90}\right|\right\}. \end{array}$$

**Figure 6.**
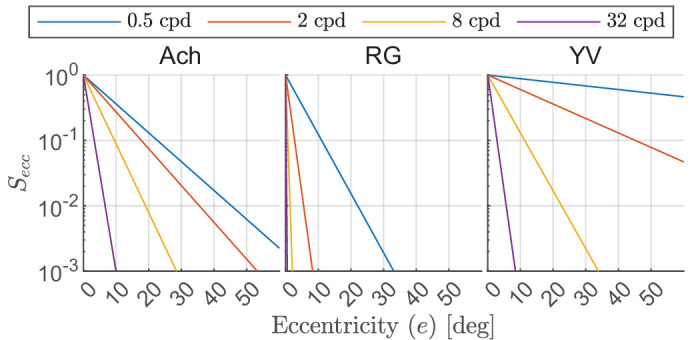
Eccentricity-dependent changes in sensitivity. The spatial frequency and the retinal orientation (axes: nasal, temporal, superior, inferior) jointly affect the rate at which the sensitivity decreases with retinal eccentricity in each color channel.

θ is the orientation in the visual field in deg. θ = 0 corresponds with the temporal and θ = 180 to the nasal directions; that is, it is an angular coordinate for the right eye. Figure 6 shows the decrease in sensitivity as the stimulus position on the retina moves from the fovea to the periphery. This decrease in sensitivity is steeper for higher spatial frequencies, which implies that our visual system is less sensitive to stimuli with finer details in the peripheral region. The slopes of eccentricity-dependent change in sensitivity also differ for the three different color channels. This is expected as the cone density function for different classes of photoreceptors (Curcio, Sloan, Kalina, & Hendrickson, 1990), as well as the sensitivity of the postreceptoral pathways for different opponent color channels (Mullen et al., 2002; Mullen et al., 2005) change at different rates in the periphery.

Another possible approach to model the effect of sensitivity could be to include eccentricity as a parameter in the critical area function in Eq. (19), as there is evidence of the critical area value increasing as a function of retinal eccentricity (Johnson et al., 1978; Wilson, 1970). However, we opted to use the simpler model from (Watson, 2018) based on the current availability of datasets. We did not find any suitable datasets that comprehensively covered the joint effects of eccentricity, area, or temporal frequency. Consequently, our model uses a simpler structure and maintains separability of eccentricity-dependence to avoid overfitting data that does not capture the joint effects of stimuli properties.

#### Combined model

The final sensitivity of our model is given by Eq. (12), which requires computing the contrast energy according to Eq. (9). The contrast energy equation relies on per-mechanism sensitivity functions, given as:

(27) $$\begin{array}{ccc} \;S_{\text{Ach}}\left(\rho ,\omega ,Y,a,e\right) & \;= & S_{\mathrm{ecc}}^{\mathrm{Ach}}\left(e,\rho \right)R_{S}^{\mathrm{Ach}}\left(\omega \right)\;S_{\mathrm{sal},S}^{\mathrm{Ach}}\left(\rho ,a,Y\right)\\ & & +\;S_{\mathrm{ecc}}^{\mathrm{Ach}}\left(e,\rho \right)R_{T}^{\mathrm{Ach}}\left(\omega \right)\;S_{\mathrm{sal},T}^{\mathrm{Ach}}\left(\rho ,a,Y\right),\; \end{array}$$

(28) $$\begin{array}{c} \;S_{\text{RG}}\left(\rho ,\omega ,Y,a,e\right)=S_{\mathrm{ecc}}^{\mathrm{RG}}\left(e,\rho \right)R_{S}^{\mathrm{RG}}\left(\omega \right)\;S_{\mathrm{sal},S}^{\mathrm{RG}}\left(\rho ,a,Y\right),\;\;\; \end{array}$$

(29) $$\begin{array}{c} \;S_{\text{YV}}\left(\rho ,\omega ,Y,a,e\right)=S_{\mathrm{ecc}}^{\mathrm{YV}}\left(e,\rho \right)R_{S}^{\mathrm{YV}}\left(\omega \right)\;S_{\mathrm{sal},S}^{\mathrm{YV}}\left(\rho ,a,Y\right),\;\;\; \end{array}$$

where S ecc c(e,ρ) is given in Eq. (25), $R_{S}^{c}\left(\omega \right)$ are the temporal filters from Eq. (13) and Eq. (14), and the combined effects of spatial frequency, stimulus area, and luminance is modelled as the product of individual sensitivities:

(30) $$\begin{array}{c} S_{\mathrm{sal},t}^{c}\left(\rho ,a,Y\right)\;=\;S_{m,t}^{c}\left(Y\right)\;S_{\mathrm{area}}^{c}\left(\rho ,a\right)\;l_{t}^{c}\left(\rho \right),\; \end{array}$$

where *c* ∈ {Ach, RG, YV}, and t∈{S,T}. $S_{m,t}^{c}$ is the luminance-dependent change in the peak sensitivity (Eqs. (21)–(23)), S area c is the function of critical area (Eq. (20)), and $l_{t}^{c}$ is the truncated log-parabola representing the CSF envelope across spatial frequencies (Eqs. (17)–(18)).

**Figure 7.**
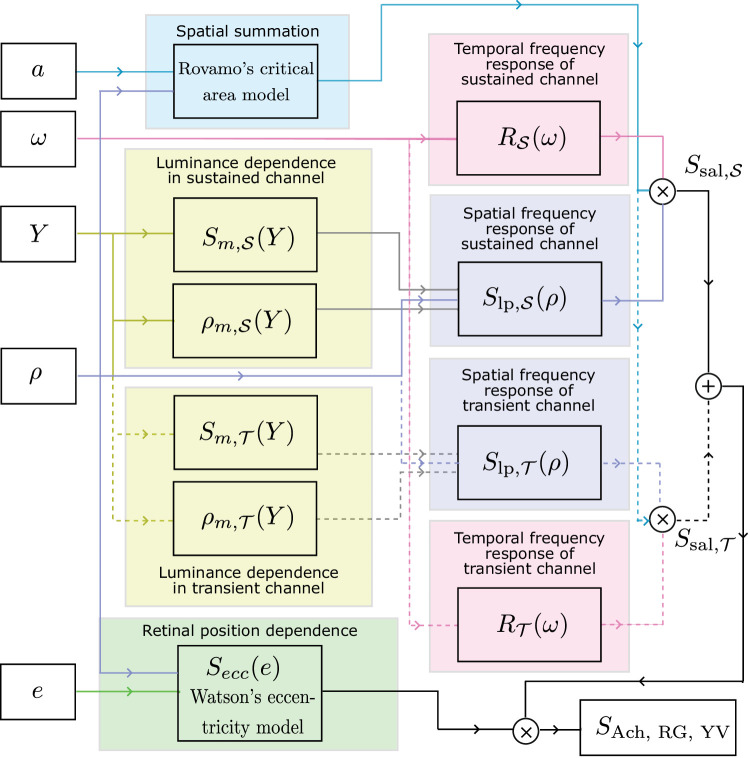
Spatiotemporal luminance, area, and eccentricity-dependent contrast sensitivity for individual opponent color mechanisms (*S*_Ach_, *S*_RG_ and *S*_YV_). The blocks represent the different components of the model described in Eqs. (16) to (29). The blocks where the inputs and outputs are dashed lines represent the transient channels and are only relevant for *S*_Ach_.

Figure 7 shows how all the individual components of our model presented in Eqs. (16) to (29) combine to predict *S*_Ach_, *S*_RG_, and *S*_YV_ which feed into our contrast encoding model (Figure 1) to predict our visual system’s sensitivity to stimuli modulated along any color direction.

#### Extension for edge stimuli

Because some of the stimuli were discs instead of Gabor patches, an extended version of our model was needed to predict them. Edge contrast sensitivity has been shown to be an indicator of the most sensitive contrast vision channel (Levi & Harwerth, 1982; Verbaken & Johnston, 1986). In other words, the peak of the contrast sensitivity envelope (across spatial frequencies) is proportional to the edge sensitivity of the visual system. Because a disc forms a circular edge, we can combine the peak-sensitivity assumption with a multiple-detectors model from (Ashraf et al., 2023) to predict the disc sensitivity as:

(31) $$\begin{array}{ccc} & & \;S_{\mathrm{disc}}^{c}\left(\omega ,Y,a,e\right)={\left(2\pi \;\sqrt{a}\right)}^{\frac{1}{\beta }}\;\max_{\rho }\left(S_{c}\left(\rho ,\omega ,Y,a_{\mathrm{disc}},e\right)\right),\; \end{array}$$

where, *c* ∈ {Ach, RG, YV}, *S*_*c*_ is the contrast sensitivity of the equivalent Gabor patches from Eqs. (27) to (29), β = 3.01142, and *a*_disc_ = 2.42437. The values of these fixed parameters are taken from (Ashraf et al., 2023).

### Model training

Eighteen datasets, listed in Tables 2–3 were used to train our CSF model. For the datasets that contained measurements from multiple observers, the mean values over all observers were used for training. All data points with sensitivity values of less than 1 were removed, because these values represent contrast thresholds greater than 1. This is only possible when asymmetric contrast modulation is employed. A per-dataset adjustment factor, *s*_*d*_, was applied to the sensitivity measurements from each dataset. This was necessary, as the absolute magnitude of the sensitivity depended on the measurement conditions, psychophysical procedure, specific stimuli used, and so on. This is compensated via a vertical shift for the whole dataset in the log sensitivity scale, allowing for the integration of diverse datasets into a single robust and generalizable model. The loss function of the optimization procedure minimized the difference between the model predictions and the adjusted (via *s*_*d*_ factor) data points from all the datasets, as well as minimized the base 10 logarithmic value of *s*_*d*_ (so the multiplier is close to 1 and prevents overfitting to any specific dataset characteristics).

(32) $$\begin{array}{ccc} & & \;L=\sum_{d}\sum_{i}{\left(\log_{10}S_{i,d}-s_{d}\log_{10}{\tilde{S}}_{i,d}\right)}^{2}+\;\frac{\lambda }{D}\sum_{d}{\left(\log_{10}s_{d}\right)}^{2}\;\;\;\;\;\; \end{array}$$

where *d* = 1, … , *D* represents the datasets and *S*_*i*,*d*_ and ${\tilde{S}}_{i,d}$ are the reference and predicted sensitivity values for the stimulus *i* in dataset *d*. We found a suitable value of the regularization parameter (λ = 0.01) by trial and error. In all our experiments, we fix *s*_*d*_ = 1 for the reference HDR CSF dataset. We fit all models using a quasi-Newton method implemented in Matlab’s *fminunc* function. To avoid local minima and implausible model parameters, the optimization was initialized using the parameters from stelaCSF (Mantiuk et al., 2022) and the post-receptoral spatiochromatic CSF (Mantiuk et al., 2020). The parameters resulting from fitting castleCSF to all available datasets are reported in Appendix Tables 5–7.

## Comparison with other CSF models

We compare the predictions of castleCSF with several popular models from the literature, listed in Table 1. We excluded from this comparison the model of Rovamo et al. (1999) because it could not be tested on our datasets (it only works only for select isoluminant directions).

One challenge of evaluating CSF models is that contrast sensitivity data cannot be easily split into datasets used for testing and training. This is because each dataset typically contains uniformly spaced samples across a few select slices of the multidimensional space of stimulus parameters. If a model is trained on one dataset and tested on another, each containing different slices of the parameter space, the error measure is not representative of the entire space. Instead of doing this, we perform a five-fold cross-validation within each dataset (leave-one-out, five splits) and use all datasets for both training and testing. The same split of test/train data was used for each compared CSF model. The error is reported as RMSE, represented in dB units:

(33) $$\begin{array}{ccc} & & \;E=20\sqrt{\frac{1}{N}\sum_{d}\sum_{i}{\left(\log_{10}S_{i,d}-s_{d}\log_{10}{\tilde{S}}_{i,d}\right)}^{2}}\;\;\left[\mathrm{dB}\right] \end{array}$$

where *N* is the total number of data points. The errors in Table 4 are reported as means and standard deviations across all five folds. The number of trainable parameters of each model is also listed in the table. Note, the parameters of the final version shown in Tables 5 to 7 are reported for the model fitted to all available data.

Because most existing CSFs model fewer stimulus parameters than castleCSF, we tested other models on the subsets of our complete dataset, shown in the columns of Table 4. For each subset, we selected only the datasets that test the dimensions modeled by the CSFs being tested and fitted each model on that subset. In particular, all the models can predict contrast sensitivity changes along spatial frequency and luminance, and so these properties were included in every comparison. The CSFs that did not model temporal frequency were tested only on static stimuli (0 Hz). Similarly, the CSFs that did not model the effect of eccentricity were tested on foveal (0° retinal eccentricity) stimuli only. In the comparisons where the effect of stimulus size was not tested, we kept only either the fixed size or the fixed cycles subsets from each dataset. The comparisons with chromatic datasets consisted of either stimulus modulated only along the cardinal color directions (*c*_*DKL*_) or modulations along any arbitrary color directions over any background color (*c*). The number of data points (*N*) included in each comparison is listed in the header of the table.

The chromatic pyramid of visibility (Watson, 2021) required special treatment because this model is not intended to predict sensitivity for low spatial and temporal frequencies. For that reason, we report the results of the test with and without (column with ρ*, ω*) (Table 4) low-frequency stimuli. We excluded the stimuli for which ρ < 4 cpd and ω < 4 Hz, unless it was isoluminant chromatic stimuli, in which case we excluded stimuli for which ρ < 1 cpd and ω < 1 Hz.

Instead of proposing a complex physiologically inspired model, we could fit a neural network to the data, which may provide an even better fit. We experimented with that idea and fitted a multilayer perceptron (MLP) using the same training/test data split as other models. The MLP had 6 layers, 120 neurons each (the architecture with the lowest validation loss). When fitted to all datasets, the error was larger than for castleCSF (5.8 dB), but more important, the MLP could not predict plausible trends or extrapolate the data. The MLP predictions can be found at the project page.^2^ This failure of general function approximations can be explained by the scarcity and non uniform distribution of contrast sensitivity data. Physiologically inspired models introduce regularization that helps them converge to plausible solutions.

castleCSF produces the lowest prediction errors for the majority of the comparisons in Table 4. The prediction error is within the range expected from inter-observer variation (between 3.0 and 3.9 dB or approximately one-half of an octave) (Dakin & Turnbull, 2016). As a reference, the expected inter observer variation for the selected datasets is listed in the rightmost column of Table 2. Notably, most CSF models result in significantly larger errors, even when tested on a much more restricted portion of the dataset. A notable exception is Mantiuk et al. (2020)’s postreceptoral spatiochromatic model, which is used as a component of castleCSF. However, this model does not cover temporal frequency and eccentricity. Overall, no existing CSF can account for the same number of stimuli parameters and provide the same accuracy of prediction produced by castleCSF.

### Model predictions

**Figure 8.**
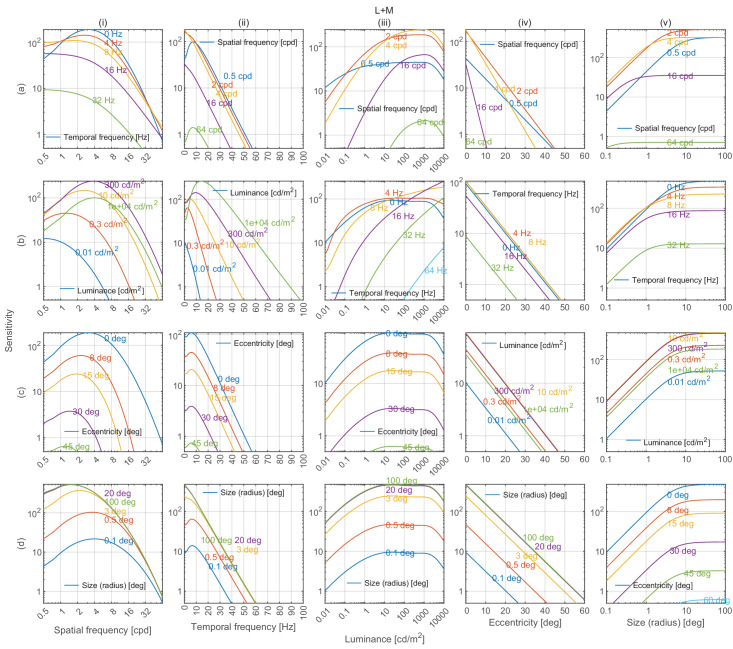
Achromatic contrast sensitivity predictions by castleCSF along five dimensions of stimuli. The unit vector of the incremental cone response (ΔL, ΔM, ΔS) of the stimuli is: (0.6612, 0.3388, 0). The contrast sensitivity response predictions are shown in the five columns with spatial frequency, temporal frequency, luminance, eccentricity, and size as independent variables respectively. In each column, the four plots show the sensitivity predictions with each of the remaining four model dimensions as the second independent variable. The remaining parameters are fixed as spatial frequency = 1 cpd, temporal frequency = 0 Hz, luminance = 30 cd/m^2^, eccentricity = 0°, stimulus size (radius) = 1°. The rows and columns are notated as a-b and i-v respectively for ease of reference in the discussion.

Cross-sections of castleCSF’s predictions for all combinations of modeled parameters, when trained on the full dataset, are shown in Figures 8 (achromatic), 9 (red-green), and 10 (yellow-violet). For each subplot, the vertical axis depicts sensitivity, and each column models changes along spatial frequency, temporal frequency, luminance, eccentricity, and stimulus size respectively as the independent variable. Within each column, rows show the joint effect of the respective independent variable and each of the remaining variables on contrast sensitivity.

**Figure 9.**
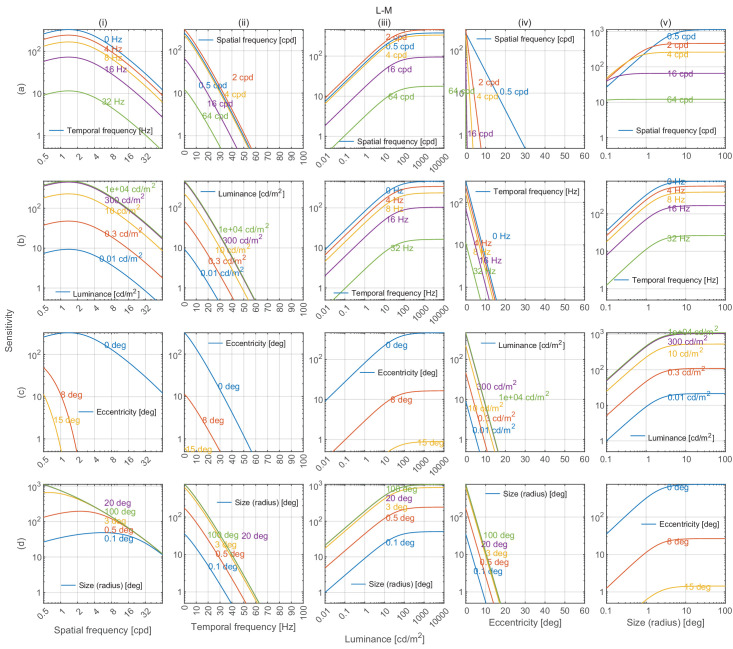
Red-green contrast sensitivity predictions by castleCSF along 5 dimensions of stimuli. The unit vector of the incremental cone response (ΔL, ΔM, ΔS) of the stimuli is: (0.3388, −0.3388, 0). The description of the plots is the same as in Figure 8.

**Figure 10.**
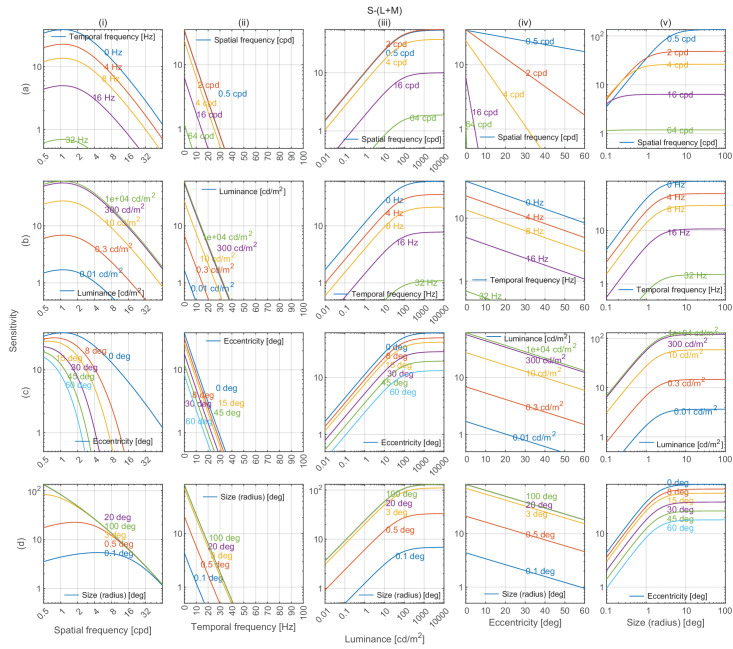
Yellow-violet contrast sensitivity predictions by castleCSF along 5 dimensions of stimuli. The unit vector of the incremental cone response (ΔL, ΔM, ΔS) of the stimuli is (0.0115, 0, 0.0115). The description of the plots is the same as in Figure 8.

**Figure 11.**
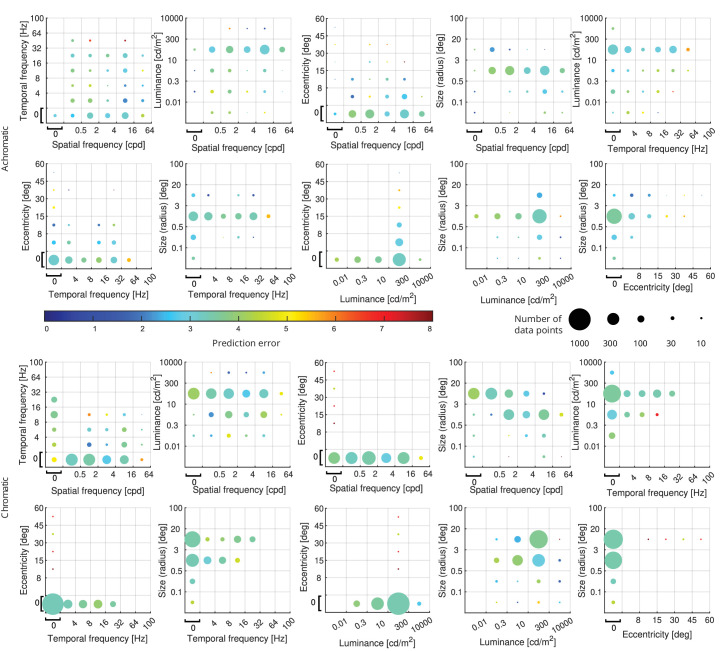
castleCSF prediction errors along with data points density for different two-dimensional projections of the five-dimensional parameter space. Top two rows: achromatic data. Lower two rows: chromatic data. The upper and lower boundaries of the bins are indicated by the corresponding tick labels on the axes. For discs, static and foveal stimuli, the whole bin represents spatial frequency = 0 cpd, temporal frequency = 0 Hz or eccentricity = 0°, respectively. The color bar in the plots represents the prediction error in decibels (dB) calculated using the error function in Eq. (33). The size of the circles represents the number of data points for each combination of parameter values.

Aggregate prediction errors for different two-dimensional cross-sections of the 5-dimensional parameter space are shown in Figure 11, along with the sample density of our combined dataset. The upper two rows in the figure represent predictions of achromatic data (*N* = 779), and the lower two show the remaining chromatic data points (*N* = 1,508). The color bar in the plots represents the prediction error in decibels (dB) calculated using the error function in Eq. (33).

## Discussion

**Figure 12.**
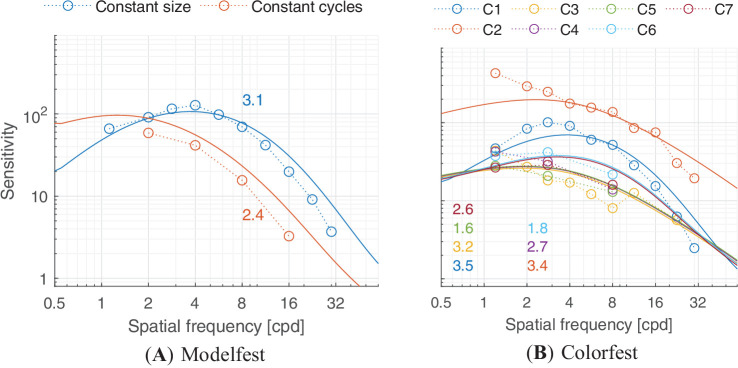
Predictions for (a) the Modelfest ((Watson, 2000)) and (b) Colorfest (Wuerger et al., 2002) dataset. (**A**) Modelfest: The stimuli were horizontally oriented achromatic static Gabor patches with either constant size (Gaussian envelope of σ = 0.5°) or constant cycles (2 visible cycles approximately) tested at 30 cd/m^2^. The viewing mode was binocular with natural pupils. (**B**) Colorfest: The stimuli were static horizontal Gabor patches of fixed size (σ = 0.5°). The color modulations were C1: black-white, C2: reddish-greenish, C3: yellowish green-violet, C4: greenish-Pink, C5: yellow-blue, C6: dark green-light pink, C7: dark yellow-light blue. C1, C2, and C3 were approximately the cardinal color directions of the human visual system. The background was D65 grey at 40 cd/m^2^. The viewing mode was binocular with natural pupils. The colored numbers shown in the plot denote the prediction error, in dB, per subset of conditions.

In this section, we discuss castleCSF predictions of individual datasets and how they relate to known psychophysical evidence. The predictions reported in this section pertain to the model fitted to the complete dataset, with the parameters listed in Tables 5 to 7 in the Appendix, using a per-dataset adjustment (*s*_*d*_ in Eq. (32)). The colored numbers shown in the Figures 12^13^,^14^,^15^,^16^,^17^,^18^,^19^,^20^,^21^,^22^,^23^,^24^,^25^,^26^ to 27 denote the fitting error for the corresponding subset of conditions, reported in dB units.

**Figure 13.**
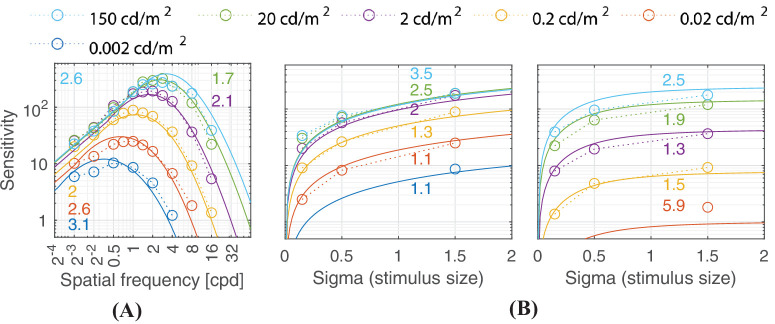
Predictions for the HDR-VDP CSF (Mantiuk et al., 2011) dataset. The stimuli in the subplot (**A**) were vertically oriented achromatic static Gabor patches with a Gaussian envelope of σ = 1.5° shown at different luminance levels. The two subplots (**B**) show measurements from similar stimuli of different sizes with spatial frequencies of 1 and 8 cpd. The viewing mode was binocular with natural pupils.

**Figure 14.**
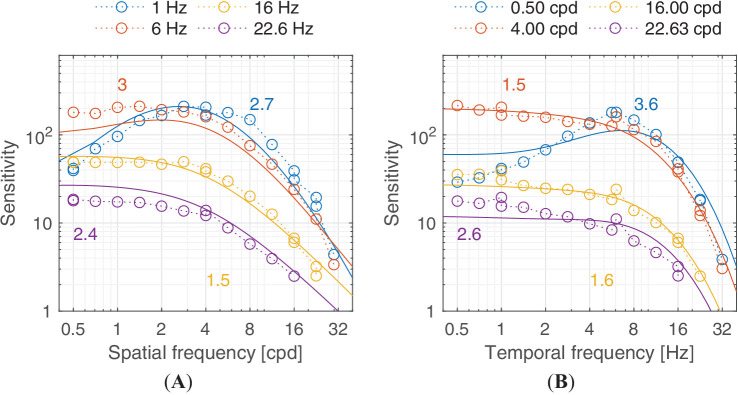
Predictions for the (Robson, 1966) dataset. The stimuli were achromatic sinusoidal gratings with rectangular apertures viewed at 20 cd/m^2^. The area of the gratings was fixed at 6.25 $\deg^{2}$.

**Figure 15.**
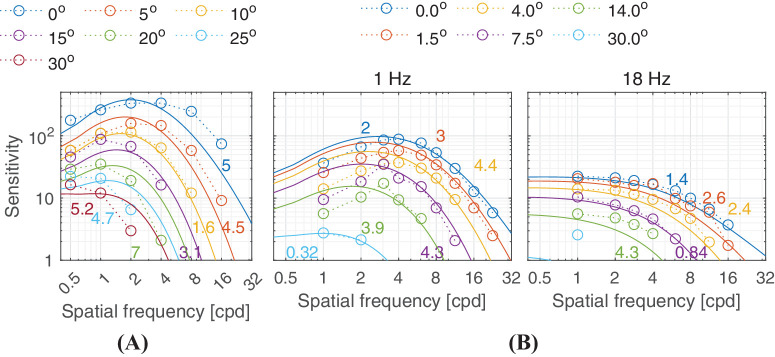
Predictions for (A) the Virsu and Rovamo (1979) and (B) Virsu et al. (1982) dataset. (**A**) The stimuli were static vertical achromatic sinusoidal gratings with circular apertures viewed at 10 cd/m^2^. The size of the aperture was fixed at a 2.5° radius. (**B**) The stimuli were horizontal achromatic sinusoidal gratings with semi-circular apertures viewed at 10 cd/m^2^. The area of the gratings was fixed at 1.57 $\deg^{2}$. The stimuli at different retinal eccentricities were moved along the horizontal retinal meridian in the nasal visual field in both datasets and the viewing mode was monocular with natural pupils.

**Figure 16.**
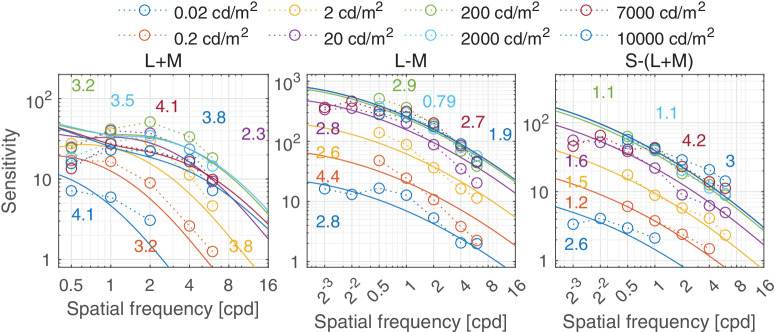
Predictions for the HDR CSF Wuerger et al. (2020) dataset. The stimuli were static vertical Gabor patches of fixed cycles (two visible cycles approximately). The three color modulations represent the cardinal color directions of the human visual system. The viewing mode was binocular with natural pupils.

**Figure 17.**
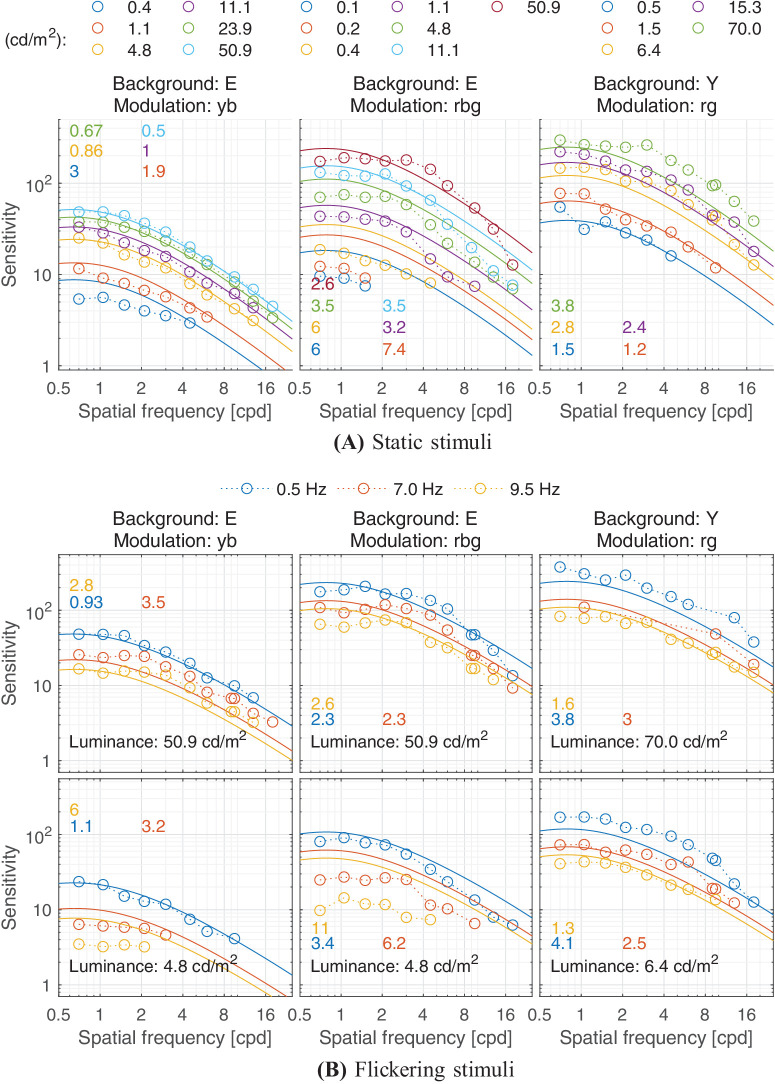
Predictions for (Van der Horst & Bouman, 1969) dataset. (**A**) Static stimuli at different luminance levels. (**B**) (Top) Temporally modulated stimuli at high luminance level. (Bottom) Temporally modulated stimuli at low luminance level. The stimuli were Gabor patches of fixed size (14.84 $\deg^{2}$). The three columns show results from the three color modulations tested: yellow-to-blue and red-to-bluish-green over grey (E: equal-energy point) background, and red-to-green over yellow (Y) background. The stimuli were viewed monocularly through an artificial pupil of 2 mm in diameter.

**Figure 18.**
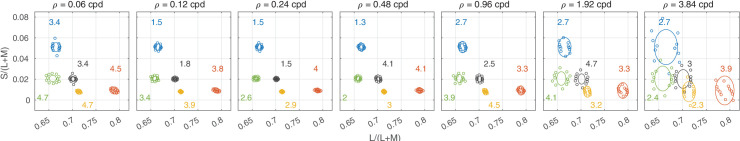
Predictions for five centers (Xu et al., 2020) dataset. Larger ellipse sizes correspond with lower sensitivity and vice versa. The stimuli were large static Gabor patches with Gaussian envelope of σ = 9.3° modulated over 6 color directions in u’v’ space on grey (72 cd/m^2^), green (24 cd/m^2^), red (14.1 cd/m^2^), blue (8.8 cd/m^2^), and yellow (50 cd/m^2^) backgrounds. The viewing mode was binocular with natural pupils.

**Figure 19.**
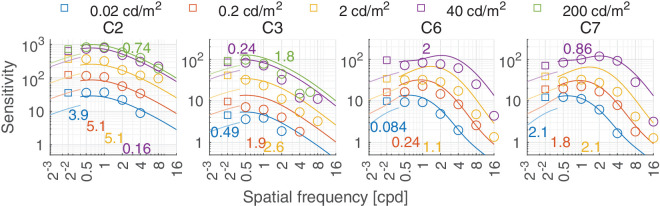
Predictions for the Kim et al. (2013) dataset. The stimuli were horizontal static Gabor patches with a Gaussian envelope of σ = 3° for the 0.25 cpd stimuli and σ = 1.5° for all other spatial frequencies shown at different luminance levels. The color modulations were C2: reddish-greenish, C3: yellowish green-violet, C6: dark green-light pink, and C7: dark yellow-light blue. C1, C2, and C3 were approximately the cardinal color directions of the human visual system. The background was D65 grey. The viewing mode was binocular with natural pupils.

**Figure 20.**
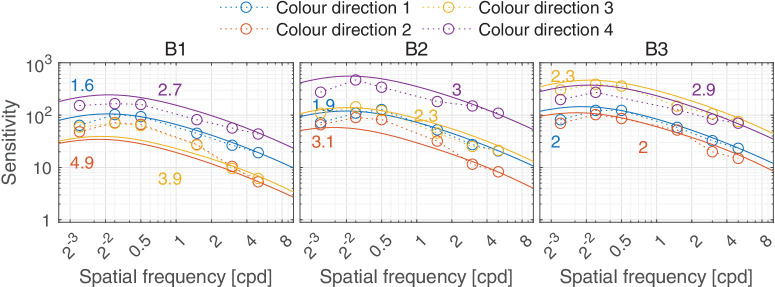
Predictions for the Lucassen et al. (2018) dataset. The stimuli were large static Gabor patches with Gaussian envelope of σ = 9.45° modulated along four color directions in u’v’ space on B1 (2600K, orange appearance), B2 (3,700K, yellow appearance), and B3 (5600, cool/daylight white appearance) backgrounds. The stimuli were shown at 108 cd/m^2^. The psychophysical task was orientation discrimination.

**Figure 21.**
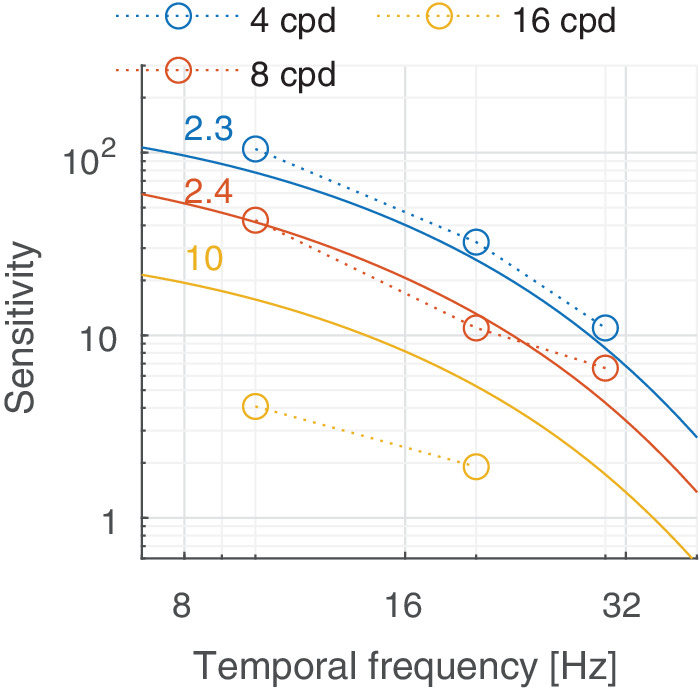
Predictions for the Laird et al. (2006) dataset. The stimuli were achromatic vertical Gabor patches of fixed size (Gaussian envelope of σ = 2.46°) displayed at 60 cd/m^2^, which were viewed binocularly with natural pupils.

**Figure 22.**
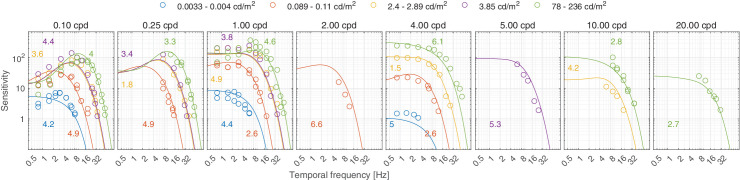
Predictions for the Snowden et al. (1995) dataset. The stimuli are achromatic Gabor patches modulated in both space and temporal domains. The Gaussian envelope size of the lower spatial frequencies up to 5 cpd is σ = 1.6°, while that of 10 cpd and 20 cpd is 0.8° and 0.4°, respectively. The lowest frequency (0.1 cpd) shown in this plot was actually a 0 cpd Gaussian blob, but we found that our model could predict this by assuming it as a Gabor patch with half a sinusoidal cycle visible. The viewing mode was binocular with natural pupils.

**Figure 23.**
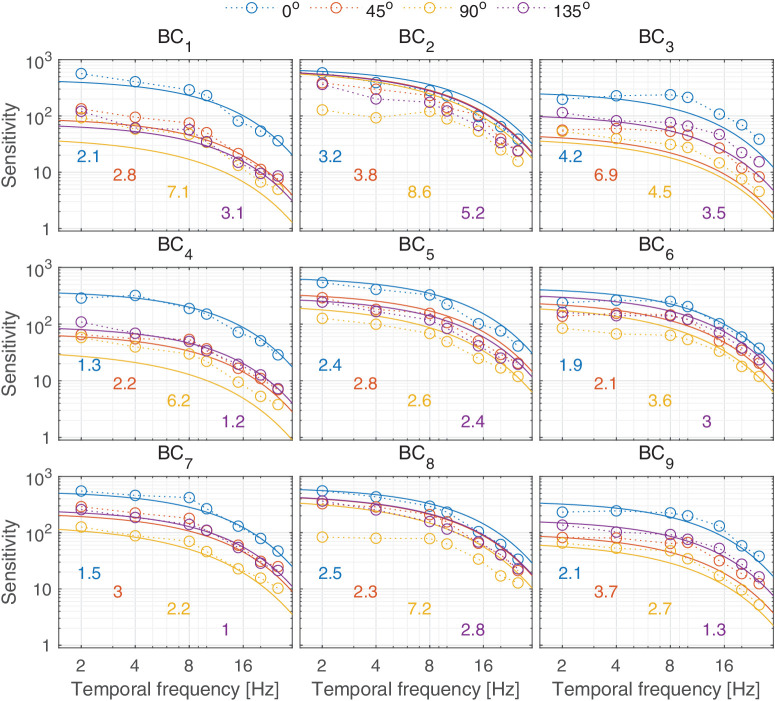
Predictions from the Kong et al. (2018) dataset. The stimuli were static discs modulated along four color directions in u’v’ space on nine different backgrounds. The approximate colors of the nine backgrounds are jade green, violet, rose red, rust orange, cornflower blue, magenta, light pink, dull violet, and dull rose red respectively. The radius of the discs was 5° and the mean luminance was 35 cd/m^2^. The viewing model was binocular with natural pupils.

**Figure 24.**
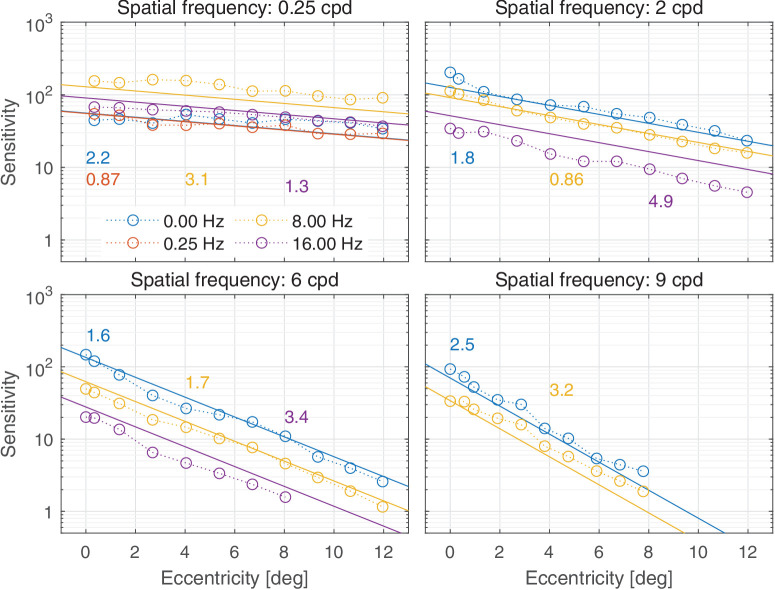
Predictions for the Wright and Johnston (1983) dataset. The stimuli were vertical achromatic sinusoidal gratings with rectangular apertures tapered at the edges viewed at 100 cd/m^2^. The area of the gratings was 36 $\deg^{2}$ for 0.25 cpd, 2.345 $\deg^{2}$ for 2 and 6 cpd stimuli, and 0.75 $\deg^{2}$ for 9 cpd stimuli. The stimuli at different retinal eccentricities were shown along the vertical retinal meridian in the superior visual field. The viewing mode was binocular with natural pupils.

**Figure 25.**
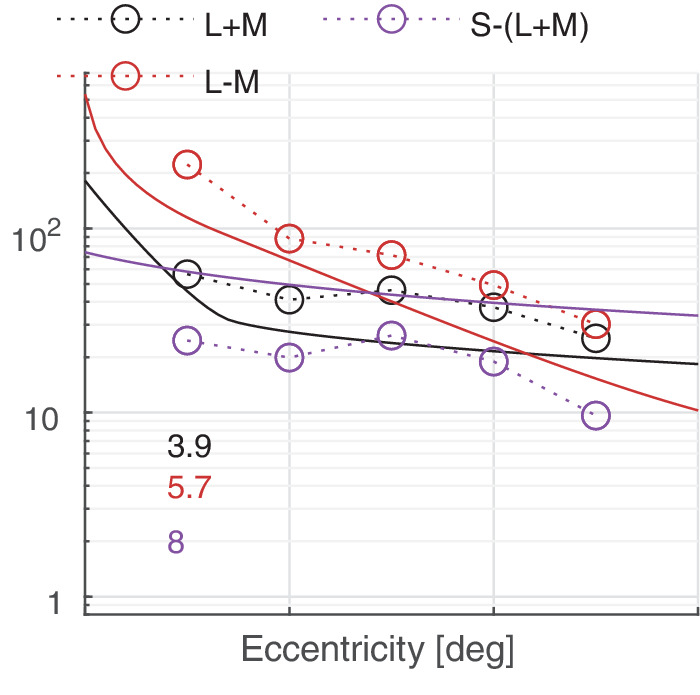
Predictions for the (Hansen et al., 2009) dataset. The stimuli were 4° diameter discs modulated along the three cardinal color directions in the DKL colorspace.

**Figure 26.**
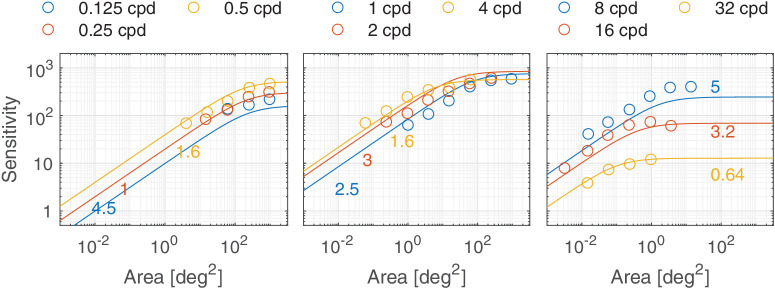
Predictions for the Rovamo et al. (1993) dataset. The stimuli were achromatic static vertical cosine gratings of different spatial frequencies with square apertures of varying areas displayed at 50 cd/m^2^. The viewing mode was binocular with natural pupils.

**Figure 27.**
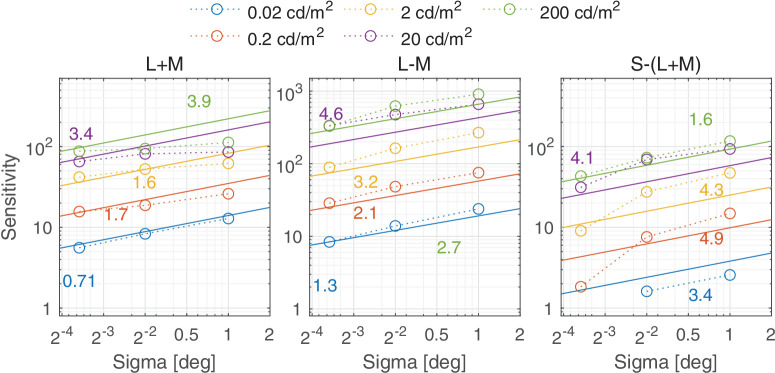
Predictions for HDR disc CSF (Ashraf et al., 2023) dataset. The stimuli were static discs modulated from a grey background to either white (L+M), pinkish red (L-M), or violet (S-(L+M)). The viewing mode was binocular with natural pupils.

### Sensitivity attenuation at high spatial frequencies

Our model predicts that contrast sensitivity decreases logarithmically with spatial frequency for both achromatic and chromatic mechanisms in the high spatial frequency region, as depicted in the plots in column (i) of Figures 8, 9, and 10. This attenuation can be observed for achromatic datasets in Figures 12A, 12B (C1), 13A, 14A, 15, and 16(L+M). The higher frequency decrement can also be observed in chromatic datasets in Figures 12B (C2-C6), 16 (L-M, and S-(L+M)), and 17 to 20.

For fixed-cycle stimuli and foveal vision, the high-spatial frequency attenuation can be solely explained by the optics of the eye (Banks, Geisler, & Bennett, 1987; Campbell & Green, 1965; Rovamo et al., 1993). Moreover, it has been shown that the slope of the MTF of the human eye changes with different pupil sizes (Deeley, Drasdo, & Charman, 1991; Van Meeteren, 1974; Watson, 2013), which in turn contract with increasing luminance levels. It follows that for fixed-cycle stimuli in natural viewing conditions, the slope of the CSF at high spatial frequencies should change with the pupil diameter and, therefore, with luminance. Unfortunately, we do not have a dataset that could demonstrate this effect (fixed-size HDR-CSF was only measured up to 6 cpd). As a result, this effect is not captured by our model, which predicts this slope to be independent of luminance level.

It is well-known that we are less sensitive to high spatial frequencies in the chromatic (isoluminant) color directions, owing to the lower resolving power of chromatic pathways (Mullen, 1985). Given that, it may come as a surprise that the ColorFest data shown in Figure 12B and HDR-CSF in Figure 16 show higher sensitivity for chromatic L-M and C2 data than for the achromatic L+M and C1 data—in particular at high spatial frequencies. This can be attributed to two issues: First, the cone contrast units, which define the sensitivity values, use an arbitrary scale for each color direction, and therefore the sensitivity values for the L+M direction cannot be directly compared to those for the L-M one, or any other. We choose the scale where the sum of (CIE, 2006) L and M cone responses is equal to the Stockman and Sharpe luminous efficiency function (Sharpe, Stockman, Jagla, & Jägle, 2005) and the resulting DKL responses reflect the length in this contrast space (Capilla, Malo, Luque, & Artigas, 1998).

In such a scale of cone fundamentals, the red-green mechanism has the strongest response (Chaparro, Stromeyer, Huang, Kronauer, & Eskew, 1993; Cole, Hine, & McIlhagga, 1993; Eskew, McLellan, & Giulianini, 1999; Kim, Reynaud, Hess, & Mullen, 2017), that is, a small change in cone contrast space would produce a large response in the red-green opponent color direction quantitatively compared to that of the achromatic or yellow-violet early vision mechanism. As a result, the perceived magnitude of contrast differs across the three dimensions of the DKL space (Switkes & Crognale, 1999). Second, neither of the two experiments attempted to isolate the chromatic mechanism of each participant (e.g., via the use of heterochromatic flicker). Therefore, the stimuli in both datasets likely contain contrast that could be detected by achromatic mechanism. For example, in Figure 12 b(C2), the spatial cut-off frequency is predicted to be at least 32 cpd, while (Mullen, 1985) estimates the cut-off for an isolated red-green mechanism to be approximately 12 cpd.

### Lateral inhibition of low spatial frequencies

Contrast sensitivity is decreased for static achromatic patterns at low spatial frequency. This effect is attributed to lateral inhibition (Barten, 1999)—the mechanism that helps us adapt to and perceive scenes spanning very large ranges of luminance. This results in a band-pass shape of the CSF for the achromatic modulation direction. When temporal frequency is increased, the effect of lateral inhibition is reduced and the CSF gradually becomes low-pass, as seen in Figure 14A and Figure 15B. (Donner & Hemilä, 1996) modeled this band-pass to low-pass spatiotemporal response as a difference-of-Gaussian receptive field model and concluded that the spatial and temporal integration mechanisms are not separable. castleCSF models this effect by gradually shifting the response from a (band-pass) sustained to a (low-pass) transient channel.

There is no evidence of lateral inhibition in the isolated chromatic mechanism (Kelly, 1983; Metha & Mullen, 1996; Mullen, 1985). However, because most of our datasets were measured for the cardinal color directions with an expected intrusion of achromatic contrast, we can see a small amount of attenuation for lower frequencies in Figure 16 (L-M, S-(L+M)) and in Figures 19 and 20.

As the eccentricity of the stimulus increases, the peak of the CSF’s band-pass shape shifts toward lower frequencies, as can be seen in Figure 15. This shift seems to be much smaller for the smaller flickering patterns used for the data in Figure 15B than for larger static patterns used for the data in Figure 15A. castleCSF does not currently model this difference.

### Temporal response

The temporal response of the visual system is mostly determined by the photoreceptors (Hood & Birch, 1993), which restrict the highest temporal frequency that can be detected. This frequency is typically modelled as the CFF, corresponding with the temporal frequency at which the sensitivity curve crosses the *S* = 1 line. We did not include in our datasets any CFF measurements—we found modelling this type of data to be highly problematic as it is typically reported for flickering disks rather than Gabors. While the generalization of Gabors and disks shown in Eq. (31) works well for static disks, we found it does not seem to extend well to flickering disks. However, our two-channel model provides a good explanation of the available sensitivity data for achromatic patterns, shown in Figure 14B, 21, and 22, and for chromatic patterns, shown in Figure 23. It should be noted that the band-pass shape and reduced sensitivity at low spatial and temporal frequencies is caused by lateral inhibition, present in the sustained channel.

### Effect of mean luminance

The luminance-dependent achromatic contrast sensitivity response can be generally divided into three regions 1) the low-luminance region following the DeVries-Rose law, 2) the mid-to-high luminance region following Weber’s law, and 3) the very high luminance region in which sensitivity is reduced. It should be noted that the categorization of the DeVries-Rose and Weber regions are merely approximations, and the measured responses are the combined result of several visual mechanisms and adaptations at work. García-Pérez and Peli (1997) and Rovamo, Näsänen, and Mustonen (1997) have discussed some nuances of using the Weber law behavior as a proxy for different physiological mechanisms.

The three different regions can be observed in plots (a-d)(iii) in Figure 8. The chromatic CSFs present only the DeVries-Rose and the Weber region as shown in plots (a-d)(iii) in Figures 9 and 10. The linear log sensitivity response with respect to log luminance obeys the DeVries-Rose law, which states that the incremental threshold contrast is proportional to the square root of the background intensity (De Vries, 1943; Rose, 1948). In the log-log scale, this square root relationship becomes linear, with an approximate slope of 0.5. With a further increase in mean luminance, this slope starts becoming flatter in accordance with Weber’s law, implying that contrast becomes independent of mean luminance (Weber, 1831). The point of transition between the DeVries-Rose region and the Weber region depends on the spatial and temporal frequencies of the stimuli. Looking at the predictions in plot (a)(iii) in Figure 8, this transition happens at lower luminances for lower spatial frequencies, and at higher luminances for higher spatial frequencies. This spatial frequency-dependent transition is also shown in the work of Rovamo et al. (1995) and is predicted well by our model, as demonstrated in analysis over datasets from HDR-VDP CSF (Mantiuk et al., 2011) (Figure 13a) and HDR-CSF (Wuerger et al., 2020) (Figure 16).

For very high luminance levels (above 2,000 cd/m^2^), our model predicts a decrease in contrast sensitivity which deviates from Weber’s law. This prediction is mainly based on the HDR-CSF dataset (Figure 16), which shows that achromatic contrast sensitivity decreases for luminance levels above 2,000 cd/m^2^. This reduction in contrast sensitivity cannot be explained by photopigment bleaching (Rovamo et al., 1997), because bleaching would proportionally reduce the response to both the background and the stimulus and thus cannot cause a reduction of retinal contrast, nor can it explain the lack of sensitivity reduction in the chromatic L-M and S-(L+M) modulation directions as the effect of bleaching would only be dependent on the luminance level and would reduce both achromatic and chromatic contrast sensitivity for the same light level. This effect cannot also be explained by increased diffraction due to the contraction of the pupil at high luminance levels, as the effect is most salient at low frequencies, which are least affected by diffraction. We speculate this decrease in sensitivity may be caused by the lateral inhibition mechanism, because it can be observed mostly at low frequencies for the achromatic mechanism, where this type of inhibition is expected to be strongest. We do not have any data to support this hypothesis, but Xin and Bloomfield (1999) have shown that the extent of lateral inhibition is dependent on the mean luminance level, with the maximum coupling between horizontal cells (and consequently the strength of contrast enhancement by lateral inhibition) occurs at moderate ambient light levels and extreme dark or light adaptation causes this effect to be weakened.

### Foveal and peripheral contrast sensitivity

Contrast sensitivity decreases with eccentricity as shown in plots (a-d)(iv) in Figures 8, 9, and 10. The slopes of these linearly decreasing functions are dependent on the spatial frequency. The rate of sensitivity decrement with retinal eccentricity is slower for lower spatial frequency stimuli compared to higher spatial frequency ones, as predicted in plots (a)(iv) for the achromatic (Figure 8) and yellow-violet (Figure 10) channels. In the case of the red-green channel (Figure 9), our model does not predict any spatial frequency dependence on the slope of sensitivity reduction with respect to retinal eccentricity. The predictions for achromatic stimuli are in line with the data from (Virsu & Rovamo, 1979) (Figure 15A) and (Virsu et al., 1982) (Figure 15B). At lower spatial frequencies, the difference between their data measured at different eccentricities is smaller, depicting a lower rate of sensitivity decline. The dataset from Wright and Johnston (1983) also shows similar findings (Figure 24) for four different spatial and temporal frequencies. The predictions for the two chromatic channels in our model are entirely driven by the Hansen et al. (2009) dataset (Figure 25). Our model could well predict the achromatic and yellow-violet stimuli from this dataset but was less accurate in predicting the red-green stimuli at higher retinal eccentricities. This could be explained by the different distribution of cells picking up red-green versus yellow-violet signals across the periphery (Mullen et al., 2002). The sensitivity of the red-green opponent mechanism also declines at a much higher rate compared to the achromatic and yellow-violet mechanisms (Mullen et al., 2002; Mullen et al., 2005). More contrast sensitivity data for chromatic stimuli measured in the periphery are required to correctly model these differences. Another interesting direction would be to add the effect of photoreceptors other than cones to predict chromatic contrast sensitivity in the periphery. Though Weale (1953) ruled out the contribution of rods in chromatic sensitivity in the periphery, Horiguchi, Winawer, Dougherty, and Wandell (2013) have shown that peripheral contrast thresholds are better explained by a four-receptor model for high-luminance stimuli and speculated that this fourth receptor could be driven by melanopsin signals. More data is needed to correctly test these hypotheses in our model.

### Spatial summation

Contrast sensitivity increases monotonically with stimulus size until a critical area is reached, following Riccó’s law (Riccó, 1877). The sensitivity remains constant for stimuli larger than this critical area as shown in plots (a-d)(v) in Figures 8, 9, and 10. The value of the critical area decreases with the spatial frequency of the stimulus for all three chromatic directions, as predicted in plots (a)(v) in Figures 8, 9, and 10. This is in agreement with the data from HDR-VDP CSF, shown in Figure 13B, and from Rovamo et al. (1993), shown in Figure 26. The HDR disc CSF dataset, shown in Figure 27 was measured for smaller sizes and thus the saturation at the critical area is not observed for these measurements. Although we lack a single dataset that would test the effect of size on the detection of chromatic patterns, the union of all datasets provides a good sampling of chromatic patterns of different sizes, as shown in Figure 11.

Note that we have a separate spatial summation model for the disc stimuli, where we integrate over the circumference of the disk (Eq. (31)). This model does not account for the likely saturation of such a summation, as we do not have data to model this effect.

Our model assumes that the effect of stimulus size is independent of temporal frequency, luminance, and eccentricity, as shown in plots (b-d)(v) in Figures 8, 9, and 10. The independence of spatial summation from luminance agrees with the data from HDR-VDP CSF as shown in Figure 13B. For both spatial frequencies, we can see that the shape of the curves formed by the data points does not change between luminance levels.

## Applications of the CSF

This section highlights engineering applications of castleCSF and shows an example in which it is used to assess the visibility of distortions caused by chroma subsampling.

**Figure 28.**
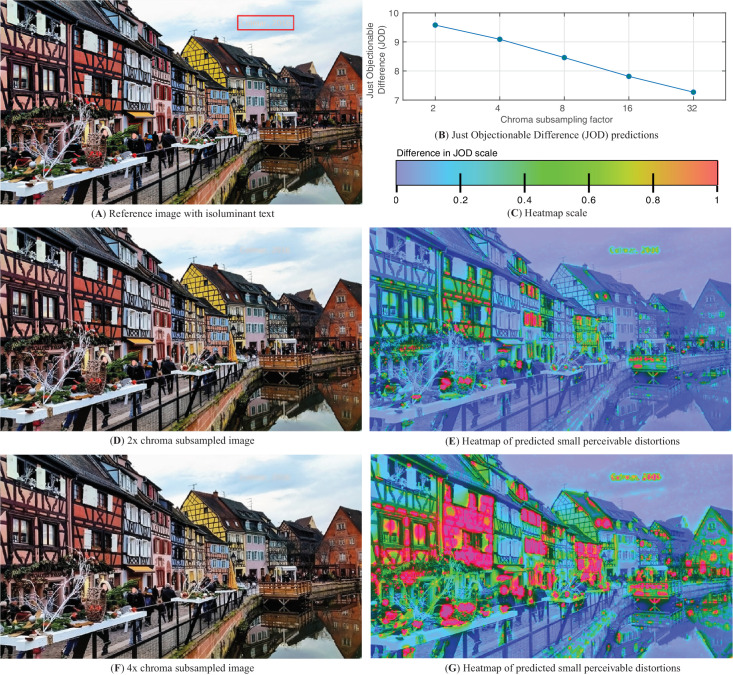
castleCSF is a fundamental building block of the ColorVideoVDP metric that can predict visible differences in complex images and videos. This figure shows a prediction of the visibility of chroma subsampling. (**A**) Reference image. (**B**) Plot of the metric’s prediction scaled in just objectionable differences (JODs) (higher values denote higher quality) against the chroma subsampling factors, showing that stronger subsampling leads to higher perceived distortion, (**D** and **F**) Chroma-subsampled versions at decreasing spatial resolutions of the chroma channels (1/2, and 1/4 respectively), (**E** and **G**) Heatmaps of predicted small (blue to red) perceivable distortions, for each subsampled image, the scale of the distortions can be seen in (**C**). Note that distortions in areas without luminance contrast (such as the text isoluminant to the sky, marked with a red box in the reference image) are especially prone to distortion from chroma subsampling. For visual assessment, these images should be viewed from a distance of 40 cm, with each image width adjusted to approximately 9 cm.

One of the main goals of creating castleCSF was to use it as a core component of the ColorVideoVDP (Mantiuk, Hanji, Ashraf, Asano, & Chapiro, 2024). This visual difference metric is used to compare a distorted image or video to its reference and predict the quality degradation due to display or coding distortions. Figure 28 shows an example in which the metric predicts the quality drop due to chroma subsampling, a computational technique commonly used in video and image compression. The effect of subsampling of both chroma channels in the CIELAB color space, the predicted visual difference maps, and the overall quality drop scaled in the just objectionable difference units (Perez-Ortiz et al., 2019) are presented. We can observe that chroma subsampling results in different levels of visible distortions across the image, with isoluminant areas, which lack luminance contrast, being the most affected. Modeling the spatiochromatic CSF, as done by castleCSF, is critical for this application.

CSF models have found many more applications in engineering. Contrast detection models were used to derive the DICOM grayscale function used in medical monitors (NEMA, 2003), and later again to define the encoding used for high-dynamic-range video standards (ITU, 2017; Mantiuk, Krawczyk, Myszkowski, & Seidel, 2004; Miller, Nezamabadi, & Daly, 2013). CSFs are used to characterize the performance of electronic displays (SID, 2022), including the visibility of flicker and display non-uniformity. Contrast sensitivity models are also employed to optimize image coding (Zeng, Daly, & Lei, 2002), match contrast visibility when reproducing high dynamic range images (Mantiuk, Daly, & Kerofsky, 2008; Tariq et al., 2023; Ward-Larson, Rushmeier, & Piatko, 1997), assess image quality (Aydin, Čadík, Myszkowski, & Seidel, 2010; Chandler, 2010; Haun & Peli, 2013; Mantiuk et al., 2011; Zhang & Wandell, 1997), or optimize rendering in real-time computer graphics (Jindal, Wolski, Myszkowski, & Mantiuk, 2021; Luebke & Hallen, 2001). Overall, given the range of applications, it is desirable to build a general model that can explain contrast sensitivity for the large range of conditions encountered in these and similar applications.

The CSF is also useful for applications in optometry, for example, the optical and neural limits of vision can be inferred by contrast sensitivity measurements (Amesbury & Schallhorn, 2003). Spatial contrast sensitivity also provides a direct measure of visual performance (Owsley & Sloane, 1987), which is very useful for clinical evaluations and for use as a diagnostic tool for the detection of visual disorders (American Academy of Opthamology, 1990).

## Conclusions

castleCSF is a practical model, intended to summarize and predict the average observer detection data for a wide range of values explored in the literature. It is mainly intended for engineering applications, in which similar models (Barten, 1999) found an ample range of use. The distinct features of castleCSF are that it explains a larger number of stimulus parameters than other models and that a single model (with the same set of parameters) can predict a broad range of measurements from the literature. Although our approach could potentially mask subtle differences between datasets that occur under specific conditions and are unique to individual datasets, we believe that the benefits of incorporating a large number of datasets outweigh the limitations considering our model’s broad application.

Our work also helps identify the gaps in the existing contrast sensitivity literature as shown in Figure 11, and can help direct future research to address these limitations. The missing datasets include edge CSF data outside the fovea, high luminance temporal CSF data for chromatic stimuli, low luminance parafoveal data, low spatial frequency chromatic temporal flicker, and data for chromatic temporal flicker in the periphery. In the case of chromatic parafoveal datasets, there is an additional challenge of representing the stimulus in cone contrast space, as the standard cone fundamentals are designed for foveal stimuli. Models of suitable modifications representing the changes in cone densities (Curcio et al., 1990; Moreland & Cruz, 1959; Volbrecht, Shrago, Schefrin, & Werner, 2000) and strength of the cone responses (Sakurai & Mullen, 2006; Stabell & Stabell, 1996) in the periphery exist. We aim to test different peripheral cone contrast metrics and the resulting peripheral opponent-color contrast channels in future iterations of this work.

## Footnotes

1 Tabulated cone fundamentals can be found at http://cvrl.ucl.ac.uk/.

2 Project page: https://www.cl.cam.ac.uk/research/rainbow/projects/castleCSF.

## Appendix: Fitted parameters

Tables 5 ^6^–7 contain the fitted parameters of castleCSF.

**Table 5.** The fitted parameters for *S*_Ach_.

Part	Parameters
Size and spatial freq.	ka,S Ach =0.1002 , kb,S Ach =0.000213, a0,S Ach =157.1, ρ0,S Ach =0.7023, ka,T Ach =0.0002412, kb,T Ach =2.676, a0,T Ach =3.816, ρ0,T Ach =3.014,
Temporal channels	βS Ach =1.331 , σS Ach =10.58, βT Ach =0.1898, σT Ach =0.08448, mω Ach =2.415, cω Ach =4.704,
Luminance	ks1,S Ach =56.49 , ks2,S Ach =7.547, ks3,S Ach =0.1445, ks4,S Ach =5.583e-7, ks5,S Ach =9.669e9, kρ1,S Ach =1.781, kρ2,S Ach =91.57, kρ3,S Ach =0.2567, ks1,T Ach =0.1934, ks2,T Ach =2748, kρ,T Ach =0.0003167,
Eccentricity	ke1 Ach =0.0189 , ke2 Ach =0.02399, ke1, nasal Ach =0.008136, ke2, nasal Ach =0.04007

**Table 6.** The fitted parameters for *S*_RG_.

Part	Parameters
Size and spatial freq.	kb,S RG =2.421 , a0,S RG =2.816442e3, ρ0,S RG =0.0711058,
Temporal channels	βS RG =1.156 , σS RG =16.43,
Luminance	ks1,S RG =681.4 , ks2,S RG =38, ks3,S RG =0.4804, kρ,S RG =0.01784,
Eccentricity	ke1 RG =2.05e-69 , ke2 RG =0.05914, ke1, nasal RG =0.1811, ke2, nasal RG =2.896e-5

**Table 7.** The fitted parameters for *S*_YV_.

Part	Parameters
Size and spatial freq.	kb,S YV =2.682 , a0,S YV =2.827890e7, ρ0,S YV =0.000635093,
Temporal channels	βS YV =0.9691 , σS YV =7.15,
Luminance	ks1,S YV =166.7 , ks2,S YV =62.9, ks3,S YV =0.4119, kρ,S YV =0.004258,
Eccentricity	ke1 YV =0.008066 , ke2 YV =0.003569, ke1, nasal YV =0.01107, ke2, nasal YV =5.858e-141
